# Secondary metabolites of *Alternaria alternate* appraisal of their SARS-CoV-2 inhibitory and anti-inflammatory potentials

**DOI:** 10.1371/journal.pone.0313616

**Published:** 2025-01-24

**Authors:** Fatma A. Moharram, Reham R. Ibrahim, Shahenda Mahgoub, Mohamed S. Abdel-Aziz, Ahmed M. Said, Hui-Chi Huang, Lo-Yun Chen, Kuei-Hung Lai, Nashwa Hashad, Mohamed S. Mady

**Affiliations:** 1 Department of Pharmacognosy, Faculty of Pharmacy, Helwan University, Cairo, Egypt; 2 Biochemistry and Molecular Biology Department, Faculty of Pharmacy, Helwan University, Cairo, Egypt; 3 Genetic Engineering and Biotechnology Division, Microbial Chemistry Department, National Research Centre, Giza, Egypt; 4 Department of Pharmaceutical Organic Chemistry, Faculty of Pharmacy, Helwan University, Cairo, Egypt; 5 School of Chinese Medicine, China Medical University, Taichung, Taiwan; 6 Graduate Institute of Pharmacognosy, College of Pharmacy, Taipei Medical University, Taipei, Taiwan; 7 PhD Program in Clinical Drug Development of Herbal Medicine, College of Pharmacy, Taipei Medical University, Taipei, Taiwan; 8 Traditional Herbal Medicine Research Center, Taipei Medical University Hospital, Taipei, Taiwan; University of Bergen: Universitetet i Bergen, NORWAY

## Abstract

This study identifies the secondary metabolites from *Alternaria alternate* and evaluates their ACE-2: Spike RBD (SARS-CoV-2) inhibitory activity confirmed *via* immunoblotting in human lung microvascular endothelial cells. In addition, their *in vitro* anti-inflammatory potential was assessed using a cell-based assay in LPS-treated RAW 264.7 macrophage cells. Two novel compounds, altenuline (**1**), phthalic acid bis (7’/7’’ pentyloxy) isohexyl ester (**2**), along with 1-deoxyrubralactone (**3**) alternariol-5-*O*-methyl ether (**4**) and alternariol (**5**) were identified. Molecular docking and *in vitro* studies showed that compounds **2** and **4** were promising to counteract SARS-CoV-2 attachment to human ACE-2. Thus, they are considered promising natural anti-viral agents. SwissADME in silico analysis was conducted to predict the drug-like potential. Immunoblotting analysis confirmed that the tested compounds (**1–4**) demonstrated downregulation of ACE-2 expression in the endothelial cells from the lungs with variable degrees. Furthermore, the tested compounds (**1–4**) showed promising anti-inflammatory activities through TNF-α: TNFR2 inhibitory activity and their inhibitory effect on the proinflammatory cytokines (TNF-α and IL-6) in LPS-stimulated monocytes. In conclusion, our study, for the first time, provides beneficial experimental confirmation for the efficiency of the *A*. *alternate* secondary metabolites for the treatment of COVID-19 as they hinder SARS-CoV-2 infection and lower inflammatory responses initiated by SARS-CoV-2. *A*. *alternate* and its metabolites are considered in developing preventative and therapeutic tactics for COVID-19.

## Introduction

Angiotensin-converting enzyme II (ACE-2) is a constitutional membrane protein found on many human cells with high expression in the vascular, heart, gastrointestinal, and type II lung alveolar cells [1]. ACE-2 is an essential part of the renin-angiotensin system (RAS) and regulates cardiac function [2]. Physiologically, ACE-2 catalyzes the conversion of angiotensin II (Ang II), a potent molecule with pro-inflammatory and vasoconstrictor properties, to Ang 1–7, thus counter-regulating the RAS through lowering Ang II [3]. Hence, working against the vasoconstrictive, pro-inflammatory, and pro-fibrotic effects of angiotensin II. Therefore, ACE-2 plays a crucial role in cardiovascular homeostasis. A critical aspect of ACE-2’s biological significance is its involvement in hypertension and heart failure. Studies have shown that decreased ACE-2 activity leads to an imbalance in the renin-angiotensin-aldosterone system (RAAS), resulting in increased levels of Ang II and subsequent vasoconstriction, oxidative stress, inflammation, and fibrosis, which are contributing factors to hypertension and heart failure.

Furthermore, ACE-2 has been linked to atherosclerosis. Its protective effects on vascular function include vasodilation, anti-inflammatory actions on endothelial cells, inhibition of smooth muscle cell proliferation, and attenuation of oxidative stress, which are essential mechanisms for preventing the development and progression of atherosclerosis. Research has shown that upregulation or activation of ACE-2 can mitigate cardiac dysfunction post-myocardial infarction by reducing inflammation and fibrosis formation within the heart tissue, leading to improved cardiac function [4–6].

ACE-2 has attracted much attention as an efficient receptor for coronavirus that results in severe acute respiratory syndrome (SARS). ACE-2 receptor is the main target of SARS-CoV-2 since it plays a vital role in the virus’s spread to alveolar cells [7]. The SARS-CoV-2 spike S1 protein—ACE-2 receptor complex is proteolytically maneuvered by type-2 transmembrane cellular serine protease (TMPRSS2) a cell surface protein mainly expressed by endothelial cells within the respiratory and digestive tracts, leading to cleavage of ACE-2 and stimulation of the spike protein, thus permitting viral entrance into the target cells. Thus, cells with ACE-2 and TMPRSS2 are more prone to viral entry [8]. ACE-2 facilitates not only the attack and rapid replication of SARS-CoV-2 but also the virus-induced damage in the membrane-bound ACE-2 protein, leading to the elevation of Ang II, which in turn triggers the release of NF-κB and pro-inflammatory cytokines [9, 10], together with the TNF-α /IL-6 pathways stimulation [11]. Thus, it ultimately results in acute damage to lung tissues [12].

Furthermore, recent findings show that NF-κB is also expressed in forming blood platelets, facilitating thrombus in COVID-19 patients [13]. Natural product has demonstrated a significant anti-inflammatory potential and offered to humanity several chemical entities that can modulate a wide range of inflammatory regulatory targets. Among these mechanisms is through interaction with intracellular signal-transducing pathways and regulation of the expression of inflammation-related genes such as nuclear factor kappa B (NF-κB), extracellular signal-regulated kinase (ERK), p38, and c-Jun N-terminal kinase (JNK) [14].

SARS-CoV-2 genome encodes for 16 and 4 non-structural structural proteins, respectively, in addition to accessory proteins [15]. SARS-CoV-2 attaches to ACE-2 for humans *via* binding of the spike (S) protein, which contains S1 and S2 subunits [16]. The subunit S1 comprises a signal peptide, C-terminal, and N-terminal domains. It is V-shaped with a receptor-binding domain (RBD), which was reported to be accountable for binding to the host ACE-2, while the S2 subunit is composed of different motifs. The fusion peptide is considered the most significant component in the viral fusion. It helps membrane fusion to the host cells, initiating a chain of conformational modifications, which reveals the cleavage of the S2 subunit, membrane fusion, and ultimately viral entry [15, 17, 18]. A computational study of FDA-approved drugs and phytochemicals from some medicinal plants against the ACE2-spike complex revealed that the five best hits from the FDA-approved database were rutin DAB10, fulvestrant, cefoperazone acid, pinaverium bromide, as well as abitrexate [19].

It was reported that ACE-2 expression is negatively connected with COVID-19 mortality [20]. Thus, ACE-2 blockers are a possible objective for antiviral intervention. Subsequently, natural product sources should be explored to find proper and potentially effective compounds against COVID-19 [21].

Moreover, the pathology of COVID-19 disease was noticed to be linked to the hyperinflammatory response referred to as a cytokine storm, which is an extreme increase in the circulating levels of pro-inflammatory cytokines including IL-1, IL-2, IL-6, IFN-γ, and TNF-α (a key role player in the cytokine storm), [22, 23], leading to ARDS (acute respiratory distress syndrome), intravascular coagulation, multiorgan failure and eventually death [24]. Recent studies reported a close relation between anti-TNF-α agents and ACE-2 inhibition. At the same time, ACE-2 /SARS-CoV -2 interaction leads to A disintegrin and metalloprotease 17 (ADAM17) activation, known as sheddase protein, or tumor necrosis factor-α-converting enzyme (TACE). The shedding activity of this protein affects the extracellular domains of other transmembrane proteins like pro-TNF-α and IL-6 receptor, causing the release of the soluble forms of IL-6 receptor and TNF-α and, subsequently, the inflammation linked to the SARS-CoV-2 infection [25]. Thus, TNF-α / IL-6R inhibitors may decrease tissue damage since this action greatly depends on creating soluble IL-6 receptors and TNF-α.

Structural studies enable the dissection of the formed complex between the ligand (small molecule) and the protein (receptor) and the identification of the critical residues responsible for its interaction. Several research groups worked diligently to determine the X-ray crystal structures of different SARS–CoV–2 parts and how mechanistically the virus can enter the human body. The most common way was through the viral surface glycoprotein (S) waltz with ACE2, allowing the virus entry [26]. Molecular docking is a computational procedure mainly used to predict the non-covalent binding of ligand-target interactions based on information derived from the knowledge of the 3D structure of a target of interest. In this study, we employed molecular docking to potentially enable guidance for anti–SARS–CoV–2 drug discovery. Additionally, a molecular dynamic simulation (MD) study was performed to validate the stability and strength of the complex formed between our compounds and the hACE2 protein. Several natural products such as polyphenolic and alkaloids have demonstrated either antagonist effects against viral entry or cell recognition through interaction with different binding receptor-binding of SARS-CoV-2 spike protein [27, 28].

*Callistemon viminalis* (weeping bottlebrush, F. Myrtaceae) is a small tree or shrub native to tropical regions such as Australia, India, and South America [29] and cultivated in Egypt. It was traditionally used to treat skin infections and gastrointestinal and respiratory disorders [30, 31]. *C*. *viminalis* contains essential oil and many bioactive metabolites such as polyphenols [32–36] and phloroglucinol [37–39]. From the biological point of view, its extract was found to exert different biological activities as cytotoxic [32, 40, 41], antioxidant [33, 34, 40–44], hepatoprotective [33], antidiabetic [45], and analgesic activities [46]. Microorganisms that live in the plant tissue in a symbiotic approach are commonly known as endophytes [47] as well as, and they represent an essential source of new and bioactive compounds with a chemical diversity structure [48]. *Alternaria alternate* (MN518330) was identified from *C*. *viminalis* leaves based on its morphological features and comparison of rDNA ITS sequence with that of *Alternaria* species. Previous reports revealed that *A*. *alternate* metabolites are considered a fungal pathogen that leads to disease for fruits and crops [49] due to the production of various phytotoxins [50]. *A*. *alternate* has several metabolites with a broad chemical structure diversity, such as steroids, terpenoids, quinones, phenolics, and nitrogen-containing compounds [51–53]. It has been stated that some of these metabolites act as mycotoxins and phytotoxins. Also, they possess various biological activities such as anticancer, antiallergic, anti-microbial, algicidal, plant growth regulation, immunomodulatory, and antimalarial [48, 52, 53].

Vaccines and drugs are considered the most consistent ways against COVID-19. Therefore, there is an urgent need to discover new drugs, especially with the incidence of mutation and genomic strains [54]. Microbes like fungi and bacteria have huge promise as a BioSource for discovering biologically active natural products, including antivirals. Furthermore, natural products make outstanding candidates for drug discovery due to their chemical diversity being more closely allied with drugs than synthetic ones; in addition, their low side effects and cost make them a rich source of biologically active agents that help in the production of different drugs [55]. Moreover, bioactive phytochemicals may become encouraging tools as adjuvants for SARS-CoV-2 infection treatment as they could assist in decreasing the inflammation in COVID-19 patients with their anti-inflammatory properties, in association with classical anti-inflammatory agents [56]. Even many natural metabolites from plants and synthetic drugs were studied for their antiviral activity but they were considerably less effective when tested in animal models. Therefore, we try to search for an alternative source of safe and economically cost-effective antiviral metabolites from microbes and study their mechanism of action, Moreover, even several metabolites and extracts of *A*. *alternate* were analysed for their different biological activity [57] but few reports about their effects for controlling COVID-19 extract, among this metabolite are alternariol and alternariol-(9)-methyl ether, which studied by different computational methods. Also its EtOAc extract and the isolated compounds exhibited antiviral activity against hepatitis C [58]. Therefore, the current study aimed to isolate secondary metabolites from *A*. *alternate* and for the first time study their effect on SARS-CoV- 2 through inhibition of ACE2/ spike RBD and controlling the inflammation. In addition, docking and molecular dynamic simulation and *in-silico* drug-like prediction were conducted to ensure safety and care.

## Methods and materials

### General

Stationary phase for column and analytical TLC, including silica gel 60, silica gel 60 F_254_, and Sephadex LH-20, were supplied from Sigma-Aldrich, Germany). Bruker Avance HD III (Bruker, Germany) was used for measuring ^1^H (400 MHz) and ^13^ CNMR (100 MHz), and the results were described as δ ppm values comparative to the internal reference (TMS) with sample concentration of 2mg/ml and operation temperature of 25°C. ESI-MS was performed on a XEVO TQD triple quadruple LC-MS. (Waters Corporation, USA). All other chemicals and solvents were obtained from El Nasr Company (Cairo, Egypt).

### Plant collection

*C*. *viminalis* (Sol. ex Gaertn.) G. Don., F. Myrtaceae) fresh leaves were obtained from the El Qanater El Khayreya botanical garden, Egypt (12/2016) at the end of the flowering stage following the local garden`s guidelines for collection and complying with the collection legislation of Egypt. *C*. *viminalis* is an ornamental tree cultivated in Egypt. Plant Authentication was recognized by Dr. Trease Labib (Mazhar Botanical Garden Egypt). The plant was deposited at the Herbarium of Pharmacognosy Department, Helwan University, Egypt (no 16 Mvi1/2016) and another plant sample was deposited at the Herbarium of El Orman Botanical Garden- El Giza-Egypt (no. 001149CC-000118-04-07-04-00118).

### Fungal isolation

Small pieces of *C*.*viminalis* leaves were washed with distilled sterilized water followed by careful treatment for the surface by 70% aqueous EtOH (two times, 2 min), then rinsed with sterilized distilled water and dried [59, 60]. The outer surface was scratched using the sterile blade, and the inner tissue was cut into small pieces, each 1cm in length, and then carefully located onto plates of PDA (potato dextrose agar) [61]. Neomycin (50 mg/L) was added to stop the growth of bacteria [62]. The plates were incubated after being sealed with Parafilm for 3–6 weeks (27°C). The resultant colonies were transported to new PDA media and retained at ≈ 4°C. Isolation of the pure strains was done by repeating the sub-culturing.

### Fungal identification

The fungal strains were identified as described in our previous work [63].

DNA sequence present in NCBI Gen Bank (http://www.ncbi.nlm.nih.gov) was used to compare the DNA sequence of the purified fungi. The phylogenetic tree was constructed using Molecular Evolutionary Genetics Analysis (MEGA, version 10.0.5) (https://www.megasoftware.net/) (**S26 Fig)**.

### Extraction of the bioactive metabolites

**Fig 1:**
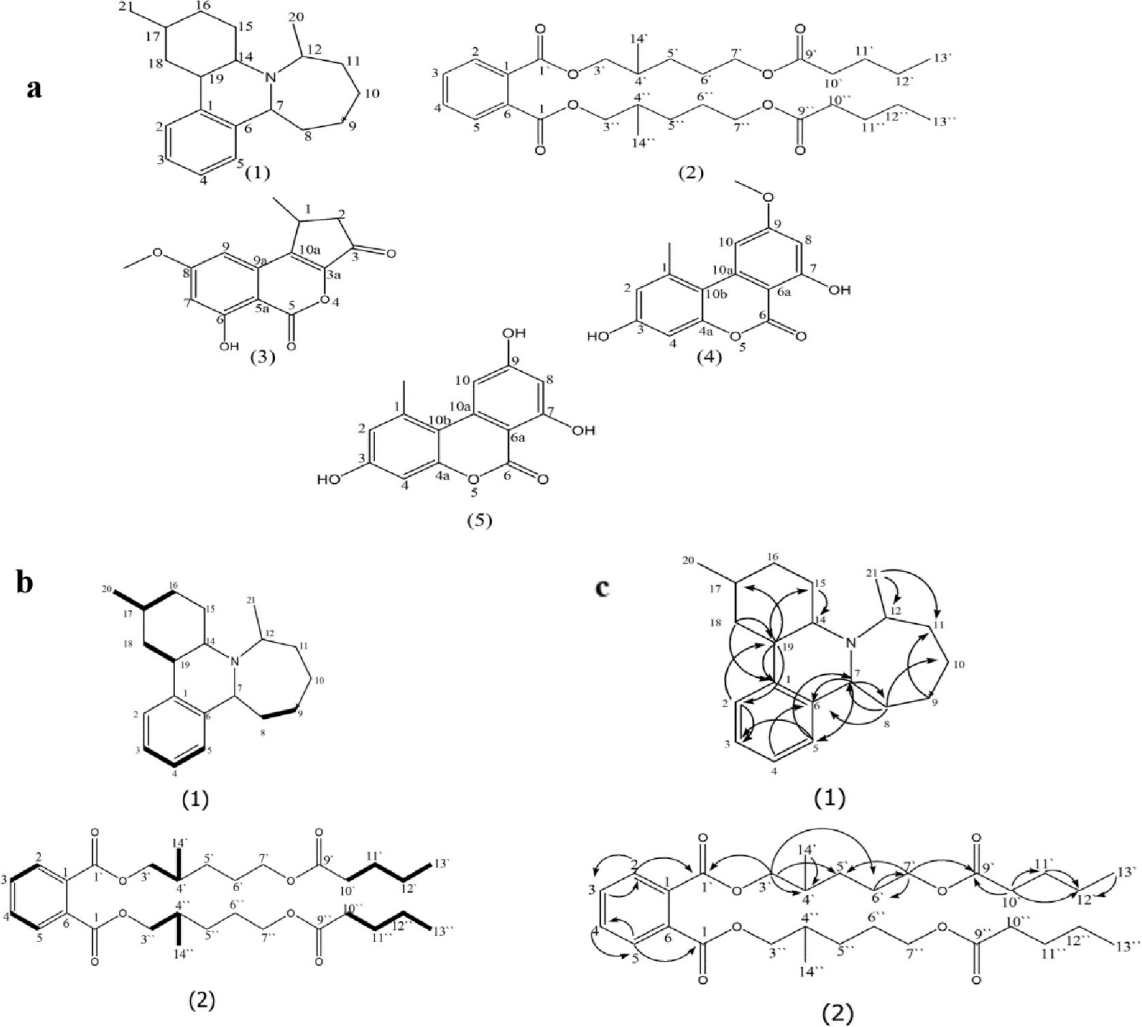
**(a**) Structures of compounds 1–5 from *A*. *alternate*. **(b)** H-HCOSY correlations of compounds 1 and 2. **(c)** HMBC correlations of compounds 1 and 2.

To prepare the mass growth of the purely cultivated fungus, it was grown on 100 g solid rice medium in Erlenmeyer flasks (10 x1 L) and then incubated at static conditions for 14 days at 30°C. After that, rice culture media was cut into small pieces and then subjected to exhaustive extraction using ethyl acetate (EtOAc), followed by evaporation till dryness (5.8 g). The EtOAc extract was fractionated on silica gel vacuum liquid chromatography (VLC) eluted by step-gradient solvent systems of *n*-hexane, petroleum ether, dichloromethane, and EtOAc to isolate the compounds. Each fraction was evaporated under reduced pressure to give 0,25, 0,5,2,0, and 0,65 for *n*-hexane. PE, DCM, and EtOAc respectively. Fractionation of PE fraction on a column of silica gel (SGC) eluted with PE / DCM (50:50 v/v) yielding pure sample of compound **1** (20 mg). DCM fraction was subjected to SGC eluted with DCM to afford three sub-fractions; 1^st^ one was further purified by SGC eluted with *n*-hexane to afford 17 mg of compound **2.** In contrast, compound **3** (15 mg) was purified from the 2^nd^ subfraction using SGC and stepwise elution using PE and EtOAc (1–0: 1–1:0:1v/v). Moreover, compound **4** (30 mg) was obtained from the 3^rd^ one by precipitation from DCM solution using excess *n*-hexane. Compound **5** (5 mg) was isolated from EtOAc fraction through SGC and gradient elution with DCM: MeOH (1:1v/v). The purity of the obtained samples was carried out depending on their color with spray reagent on TLC and appearance under UV light [64] (**Fig 1**).

**Compound 1:** Colorless oil; ^1^HNMR, CDCl_3_ (400 MHz), and ^13^CNMR (100 MHz) results are represented in **Table 1**). +ve ESI/MS, *m/z* 284.3294 [M+H] ^+^ (calcd for C_20_H_29_N). 1 and 2D HNMR and ESI-MS spectrum are represented in supplementary information (**S1–S7 Figs)**.

**Table 1:** ^1^H and ^13^C NMR data for compound 1 (CDCl_3,_ 400 and 100 MHz).

No	δ_C_	δ_H_* (mult; J = Hz)	HMBC	APT	COSY
**1**	148.0			Q	
**2**	127.1	7.36 (m)	C-19, C-3	CH	
**3**	125.8	7.33 (m)		CH	H-4
**4**	128.3	7.44 (m)	C-6	CH	H-3
**5**	127.8	7.32 (m)	C-3, C-7	CH	H-4
**6**	146.5			Q	
**7**	46.3	2.67 (br d)	C-5, C-6, C-8	CH	
**8**	37.2	1.79 (m)	C-6, C-7, C-10		H-9
**9**	27.8	1.35 (m)	C-11		H-8
**10**	20.9	1.36 (m)			
**11**	29.9	1.87–1.7			
**12**	48.1	2.58 (m)		CH	
**14**	46.0	2.34 (m)		CH	
**15**	38.7	1.76 (m)	C-14		
**16**	22.8	1.06 (m)	C-17, C-18		H-17
**17**	22.7	1.43 (m)	C-16		H-20
**18**	32.1	1.42 (m)	C-1, C-19		H-19
**19**	40.2	2.86 (m)	C-1, C-2, C-15, C-17	CH	H-18
**20**	14.2	1.05 (br	C-17	CH_3_	
**21**	12.3	0.96 (br d)	C-11, C-12	CH_3_	

*Assignment of exact proton signals with its carbon are obtained from HMQC spectrum.

**Compound 2:** Faint yellow viscous oil; ^1^HNMR CDCl_3_ (400 MHz, CDCl_3_) and ^13^CNMR (100 MHz) data see **Table 2**. negative ESI-MS, *m/z* at 533.1912 [M- H]^-^ (calcd, for C_30_H_46_O_8_). 1 and 2D HNMR and ESI-MS spectrum are represented in (**S8–S14 Figs)**.

**Table 2:** ^1^H and ^13^ CNMR data for compound 2 (CDCl_3,_ 400 and 100 MHz).

No	δ_C_	δ_H_* (mult; J = Hz)	HMBC	APT	COSY
**1/6**	132.5			Q	
**2/5**	128.8	7.72 (br d)	C-3/4,1`/1”	CH	H-3/4
**3/4**	130.9	7.54 (br d)	C-2/5	CH	H-2/5
**1`/1”**	167.7			Q	
**3`/3”**	68.2	4.24 (m)	C-1`/1”, C-4`/4”, C-5`/5”, C-6`/6”	CH_2_	H-4`/4”
**4`/4”**	38.7	1.70 (m)	C-3`/3”	CH	H-3`/3”
**5`/5”**	30.4	1.35 (m)		CH_2_	
**6`/6”**	23.8	1.37–1.42 (m)	C-7`/7”	CH_2_	
**7`/7”**	66.7	3.99 (m)	C-5`/5”, C-6`/6”,C-9`/9”	CH_2_	
**9`/9”**	174.0			Q	
**10`/10”**	34.4	2.31 (t, 7.2)	C-9`/9”C-11`/11”, C-12`/12”	CH_2_	H-11`/11”
**11`/11”**	25.0	1.63 (m)	C-12`/12”	CH_2_	H-10`/10”, 12`/12”
**12`/12”**	29.1	1.32 (m)		CH_2_	H-11`/11”, 13`/13”
**13`/13”**	10.9	0.93 (m)	C-12`/12”	CH_3_	H-12`/12”
**14`/14”**	14.0	0.91 (d)	C-4`/4”	CH_3_	

*Assignment of exact proton signals with its carbon are obtained from HMQC spectrum.

**Compound 3** was isolated as a colorless amorphous powder; negative ESI/MS, *m/z* 259.1458 [M- H]^-^ (calcd for C_14_H_12_O_5_). ^1^HNMR (400 MHz, CDCl_3_), δ_H_ 11.36 (s, 6-OH), 6.72 (1H, brs, H-9), 6.71 (1H, brs, H-7), 3.96 (3H,s, 8-OCH_3_); 3.45 (1H, m, H-1**)**, 2.99 (1H, dd, 1.4, 19.9, H-2_a_), 2.35 (1H, dd, 0.4, 18.4, H-2_b_), 1.48 (3H, d, J = 6.8, 1-CH_3_); ^13^CNMR (100 MHz, CDCl_3_) data δ_C_ 95.2.1 (C-3), 166.8 (C-8), 165.3 (C-6), 164.9 (C-5), 148.2 (C-3_a_), 144.6 (C-10), 134.5 (C-9_a_), 103.06 (C-9), 100.9(C-5_a_), 56.03 (8-OCH_3_),), 42.8 (C-2), 28.4 (C-1), 21.0 (1-CH_3_). Spectroscopic results are represented in (**S15–S20 Figs)**.

**Compounds 4** and **5** were isolated as reddish-white needles as well as displaying blue fluorescence with long UV light (360 nm). They were identified as alternariol-5-O-methyl ether (**4**) and alternariol (**5**) based on a comparison of their ^1^H NMR and negative ESI-MS with previously published data [33]. The 1D HNMR and ESI spectrums are represented in (**S21–S25 Figs).**

### In silico evaluation using docking studies

The in-silico analysis for the isolated metabolites to block ACE-2-SARS-CoV-2 Spike RBD binding site was performed by evaluating the binding of the isolated compounds as well as a known natural inhibitor, quercetin, inside the ACE-2-SARS-CoV-2 Spike RBD binding site. The crystal structure of the SARS-CoV-2 spike receptor binding domain complexed with ACE-2 (PDB: 6LZG) was used. The 3D structures of the endophyte metabolites (1–4) were achieved by the software of Discovery Studio (Accelrys Inc., USA). AutoDock Vina (Scripps Research, California, USA) [65] prepared the receptor and ligands as pdbqt files after eliminating water, adding polar hydrogen atoms, and Gasteiger charges, respectively. AutoDock Vina uses a Monte-Carlo iterated local search method involving iterations of sampling, scoring, and optimization. The docking parameters were as follows: grid box size (34x42x54 Å^3^) was adjusted to encompass the interaction site. An exhaustive value of 8 and an energy range value of 4 were used while keeping the other parameters with their default values. The parameters for docking were first validated by redocking the already bound ligand co-crystallized with the protein. The binding mode obtained matches that of the crystal structure; if a closely similar binding mode is received, the parameters are used for the docking studies. The most stable docking pose attained was nominated for comparing the binding mode. Visualization of ligand-protein non-covalent interactions, as well as schematic 2-D illustrations of enzyme-ligand complexes, was accomplished by the software Discovery Studio.

### Molecular dynamic simulation method

In this study, molecular dynamic simulations (MDS) were conducted for 100ns using GROMACS 2.1.1 software [66] on compound **2** and compound **4**. The retrieved docking coordinates of ACE2 bound to compound 2 and compound 4 were used as input structures for molecular dynamics. The receptor and ligand topologies were generated by PDB2gmx (embedded in GROMACS) and GlycoBioChem PRODRG2 Server respectively, both under GROMOS96 force field. After rejoining ligands and receptor topologies to generate the system, the typical molecular dynamics scheme of GROMACS was applied for all the systems. This includes solvation, neutralization, energy minimization under GROMOS96 43a1 force field and two stages of equilibration (NVT and NPT). Finally, an unrestricted production stage of 100ns was applied for the four systems with particle mesh ewald (PME) method implemented to compute the long-range electrostatic values using 12 Å cut-off and 12 Å Fourier spacing. The first stage is to judge the stability of the complexes using RMSD and RMSF values calculated from the MDS trajectories from the production step. The second stage is performing MM-PBSA calculation and per residue contribution. The MM-PBSA package of Kumari *et al*. [67] was contrived to calculate the binding free energy between the ligands and the two receptors using the following equation.


$$\Delta G_{\left(Binding\right)}=G_{\left(Complex\right)}-G_{\left(Receptor\right)}-G_{\left(Ligand\right)}$$

All formed complexes were subjected to such calculations.

### *In-silico* pharmacokinetic analyses

We have used the open access *in silico* prediction tools for predicting the in silico ADMET properties of compounds (1–5) included in this study (Details are included in the supplementary materials) The available bioinformatics open access SwissADME (http://www.swissadme.ch/index.php) [67] was used for predicting the drug-likeness attributes. Lipinski’s rule of five was used to analyze the properties such as hydrogen bond donor (HBD), hydrogen bond acceptor (HBA), molecular weight (MW), topological polar surface area (TPSA) and lipophilicity (log P) and PreADMET (https://preadmet.bmdrc.kr/) [68] was used to determine the toxicity attributes of selected compounds included in this study.

### Biological evaluation

#### In vitro ACE-2: Spike RBD (SARS-CoV-2) inhibitor screening colorimetric assay

ACE-2: SARS-CoV-2 Spike (RBD), inhibitor screening colorimetric assay kit (Bps Bioscience, Cornerstone Court W, Ste B San Diego, CA 92121) was employed [69, 70] for evaluating the activity of compounds **1**–**4** and EtOAc extract of *A*. *alternate* with the guide of the manufacturer’s directions. Different concentrations of EtOAc extract (0.038–9.60 μg/mL) and compounds **1**–**4** (1.25–50 μM) or quercetin as a positive control [71, 72] were utilized.

#### Cultured cells & cell viability assay

Human lung microvascular endothelial cells (HLMEC) were acquired from Lonza (Hayward, CA, USA). The cells were seeded in an endothelial cell growth medium (EGM-2 MV) (Lonza, Hayward, CA, USA) at 37°C and 5% CO_2_. Then, at 5 to 7 passages, the cells were plated in 100 μL/well EGM-2 MV medium in collagen-covered 96-well plates. The cells were grown for 3–5 days to reach the confluent layer. Cell viability was valued by the MTT Cell Growth Assay Kit (Sigma-Aldrich, Steinheim, Germany) [73] using untreated cells as control.

#### Determination of ACE-2 protein using western blot

HLMEC cells were seeded, cultured, and incubated with compounds **1–4** for 48h. RIPA buffer (Cell Signaling, Danvers, MA) was employed to obtain the entire-cell protein lysates. Bradford method [74] was used to determine the concentration of protein. Western blot assay was done as previously stated [75] using untreated cells as control. Anti-ACE-2 antibody [EPR24705-45] ab272500 (Abcam, Cambridge, UK) was used.

#### Evaluation of anti-inflammatory activity

*In vitro TNF-α [Biotinylated] inhibition assay*. TNFR2: TNF-α [Biotinylated] inhibitor screening assay kit (BPS bioscience, Cornerstone Court W, Ste B San Diego, CA 92121) was used to screen for the *in vitro* inhibition of TNF-α binding to tumor necrosis factor receptor 2 (TNFR2) following the manufacturer’s protocol [76, 77]. Different concentrations of EtOAc extract (0.48–7.60μg/mL), compounds **1–4** (0.25–64μM), or certolizumab as a positive control were utilized.

*Evaluation of pro-inflammatory cytokines (TNF- α and IL-6) using LPS-induced RAW 264*.*7 cells*. RAW 264.7 macrophage cells were procured from the American Type Culture Collection (Manassas, VA, USA) and grown as previously described. *In vitro* Toxicology Assay Kit, MTT Based (Sigma-Aldrich, Saint Louis, Missouri, USA) was employed to establish cell viability at concentrations (1–250 μM). Treatment of RAW 264.7 cells and determination of the inhibitory effect of the active compounds at sub IC_50_ values on TNF- α and IL-6 gene expression in RAW264.7 cells were performed as mentioned before [78] using LPs treated cells as control. *TNF- α*, *IL-6*, and the housekeeping gene ACTB*-*specific RT primers are demonstrated in **(S1 Table)**.

### Statistical analysis

The entire experimental records were obtained from three distinct experiments accomplished in trio. p values < 0.05 were considered significant. Statistical significance was obtained by One-Way Analysis of Variance (ANOVA) with post hoc Tukey via GraphPad Prism 5.0 (Graph Pad Inc., USA) as well as non-linear regression analysis for determination of IC_50_ values.

## Results

### Molecular identification of the isolated endophytic fungus and compounds

The isolated fungus was identified as *Alternaria alternate* after comparing its 18SrRNA nucleotide sequence by the NCBI database, which exhibits 100% similarity with each other. It was deposited in Gen Bank with accession number MN518330, and its phylogenic tree is represented in **(S26 Fig).** Moreover, it is preserved in Egypt Microbiological Culture Collection (EMCC) with an EMCC number 28558 on 29-3-2023.

Five compounds (**1–5**), (**Fig 1**) were isolated from EtOAc extract among which two new compounds (**1** and **2)**, in addition to three known compounds **3**, **4**, and **5,** identified as 1-deoxyrubralactone (**3**), alternariol-5-*O*-methyl ether(**4**), and alternariol (**5**) based on the comparison of their NMR and MS data with reported data [33, 79, 80].

**Compound 1** was obtained in pure form as a colorless oil with molecular formula C_20_H_29_N. It displayed a molecular ion peak at *m/z* 284.3294 [M +H]^+^, indicating the presence of an odd number of N-atoms in the structure as well as the presence of seven degrees of unsaturation, which confirmed the presence of three double bonds and four rings in the structure. ^1^HNMR spectrum displayed an unresolved aromatic system due to *ortho-* and *meta-*couplings at δ_H_ 7.44, 7.36, 7.33, and 7.32 for H-4, H-2, H-3, and H-5, respectively. Moreover, in the aliphatic area, five groups of protons establish the occurrence of five methine groups at δ_H_ 2.86, 2.67, 2.58, 2.34, and 1.43 for H-19, H-7, H-12, H-14, and H-17, respectively as well as, their downfield shift due to their position near to aromatic ring and a nitrogen atom. Moreover, a complex overlapping multiplet hydrogen proton signals for several methylene groups present in the same chemical environment. In addition, there are two broad doublet signals for the two-methyl groups CH_3_-20 (δ_H_ 1.05) and 21 (δ_H_ 0.96). ^13^CNMR spectrum showed 20 carbons, among which four aromatic methine groups (δ_C_ 125.8–128.3), five aliphatic carbons (δ_C_ 22.7–48.1), seven methylenes (δ_C_ 20.9–38.7), two methyl (δ_C_ 12.3 and 14.2) and two quaternary aromatic carbons. (δ_C_ 146.0–148.0). The three methine groups attached to the nitrogen atom were confirmed by their downfield shift at δ_C_ 48.1,46.3,46.0 for CH-12, 7, and 14, respectively. This evidence was established by HMQC and APT experiments. Moreover, the H-HCOSY (**Fig 1**) showed that the correlation between H-3 and H-5 with H-4 established the presence of an aromatic ring, and the correlation between H-17 and CH_3_-20 placed them at C-17. Moreover HMBC (**Fig 1**) confirmed the structure of **1** through the correlation of the aromatic H-2 (δ_H_ 7.36) with C-3 (δ_C_ 125.8) and C-19 (δ_C_ 40.2), H-4 (δ_H_ 7.44) with C-6 (δ_C_ 146.5) and that from H- 5 (δ_H_ 7.32) to C-3 (δ_C_ 125.8) and C-7 (δ_C_ 46.3), which confirmed aromatic ring configuration and the connectivity between it and the side chain at C-2 and 6. The structure of the saturated rings and their connectivity were established through the correlation between different carbons and protons signals, among which the correlation from H-7 (δ_H_ 2.67) and C-5 (δ_C_ 127.8), C-6 (δ_C_ 146.5), C-8(δ_C_ 37.2) and that between CH_2_-8 (δ_H_ 1.79), C-6 (δ_C_ 146.5), C-7(δ_C_ 46.3) and C-10 (δ_C_ 20.9); CH_2_-9 (δ_H_ 1.35) with C-11(δ_C_ 29.9); CH_2_-15 (δ_H_ 1.76) and C-14 (δ_C_ 46.0); CH_2_−16 (δ_H_ 1.06) with C-17 (δ_C_ 22.7), and C-18 (δ_C_ 32.1), CH_2_-18 (δ_H_ 1.42) with C-1(δ_C_ 148.0), C-19 (δ_C_ 40.2). The connectivity of the CH_3_-20 at C-17 was confirmed by a correlation between it and C-17 (δ_C_ 22.7), as well as the correlation from CH_3_-21 to C- 11 (δ C 29.9) and C-12 (δC 48.1), established its position. Therefore, compound **1** was identified as altenuline, which was not identified from nature before.

**Compound 2** was purified as faint yellow viscous oil; it was soluble in DCM and insoluble in water, indicating its low polarity. ^1^HNMR spectrum revealed the occurrence of two signals (br d); at δ_H_ 7.72 and 7.54 for H-2/5 and 3/4, respectively which confirm the occurrence of di *ortho*-substituted aromatic ring. The characteristic 3’/3’’ and 7’/7’’ methylene group attached to O-C = O group was established from the multiplet signal at δ_H_ 4.24 and 3.99, respectively integrated for four protons, as well as methylene group at C10’/10’’ appeared as triplet signal at δ_H_ 2.31 due to its attachment to C = O. Furthermore, a multiplet signal at δ_H_ 1.70 revealed the presence of a methine group (CH- 4’/4’’) bounded to two methylene groups. In addition, there were complex multiplet overlapping signals for several hydrogen protons characteristic for different methylene groups laying in the same chemical environment (H- 5’/5’’, H-6’/6’’, H-11’/11’’ and 12’/12’’). Furthermore, ^1^HNMR data revealed the presence of up-field multiplet signals and broad doublet signal at δ_H_ 0.93 and 0.91, indicative of two terminal methyl groups_,_ CH_3_-13’/13’’ and 14’/14’’, respectively. ^13^CNMR, HMQC, and APT spectrum showed the occurrence of six aromatic carbons, two of them were quaternary carbons C-1/6 (132.5) and four methine groups, C-2/5 (128.8), C-3/4 (130.9). In addition to two carbonyl carbons, one of which (C-1’/1’’ δ_C_ 167.7) is upfield due to its attachment to the aromatic ring while the other is downfield (C-9’/9’’, δ_C_ 174.0). The downfield shift of the two-methylene groups 3’/3’’ (δ_C_, 68.2) and 7’/7’’ (δ_C_ 66.7) confirm their attachment to O-C = O. Moreover, the structure contained only one methine group at δ_C_ 38.7 (CH- 4’/4’’) and two methyl groups CH_3_-13’/13’’ and 14’/14’’ at δ_C_ 10.9 and 14.0, respectively. Furthermore, there were another five CH_2_ groups at δ_C_ 30.4, 23.7, 34.4, 25.0, and 29.1 for C-5’, 6’, 10’,11’, and 12’, respectively. H-H-COSY (**Fig 1**) established the correlation between the aromatic ring’s protons and that between the side chains’ protons. Additionally, HMBC data (**Fig 1**) confirmed the substitution of the aromatic ring through the correlation from H-2/5 (δ_C_ 7.72) to C-3/4 (δ_C_ 130.9) and that between H-3/4 (δ_C_ 7.54) and C-2/5 (δ_C_ 128.8). The connectivity of the side chain to the aromatic ring at C-1 and C-6 was established through the correlation from H-2/5 (δ_H_ 7.72) to C-1`/1” (δ_C_ 167.7). Moreover, the correlation from H 3`/3” (δ_H_ 4.24) and C-1`/1” (δ_C_ 167.7), C-4`/4”(δ_C_ 38.7), C-5`/5” (δ_C_,30.4) and C-6`/6” (δ_C_ 23.8) as well as that from H 7`/7” (δ_H_ 3.99) and C-5`/5”(δ_C_ 30.4), C-6`/6” (δ_C_ 23.8) and C-9`/9” (δ_C_ 174.0) gave evidence for their attachment to O-C = O. Furthermore, the connectivity of methyl group at C-14`/14”and C-13`/13”was confirmed by the correlation from H-14`/14” (δ_H_ 0.91) to C-4`/4” (δ_C_ 38.7) and that between H-13`/13” (δ_H_ 0.93) with C-12`/12” (δ_C_ 29.1). The other correlations which confirmed the structure were listed. Despite ^13^CNMR displaying only six aromatic carbons and twelve carbons among which two carbonyl carbons, one methine, seven methylene, and two methyl groups, the -ve ESI/MS of **2** showed *m/z* at 533.1912 [M- H]^-^ which established the presence of two identical mirror image parts and considered a symmetrical dimer of 1,6 –benzene dicarboxylic acid thus, identified as phthalic acid bis (7’/7’’ pentyloxy) isohexyl ester. Based on our search in Science Finder, compound **2** is considered a new natural product.

### In silico analysis for the identified metabolites against hACE-2 and SARS-CoV-2 spike RBD)-ACE-2 complex

**Fig 2:**
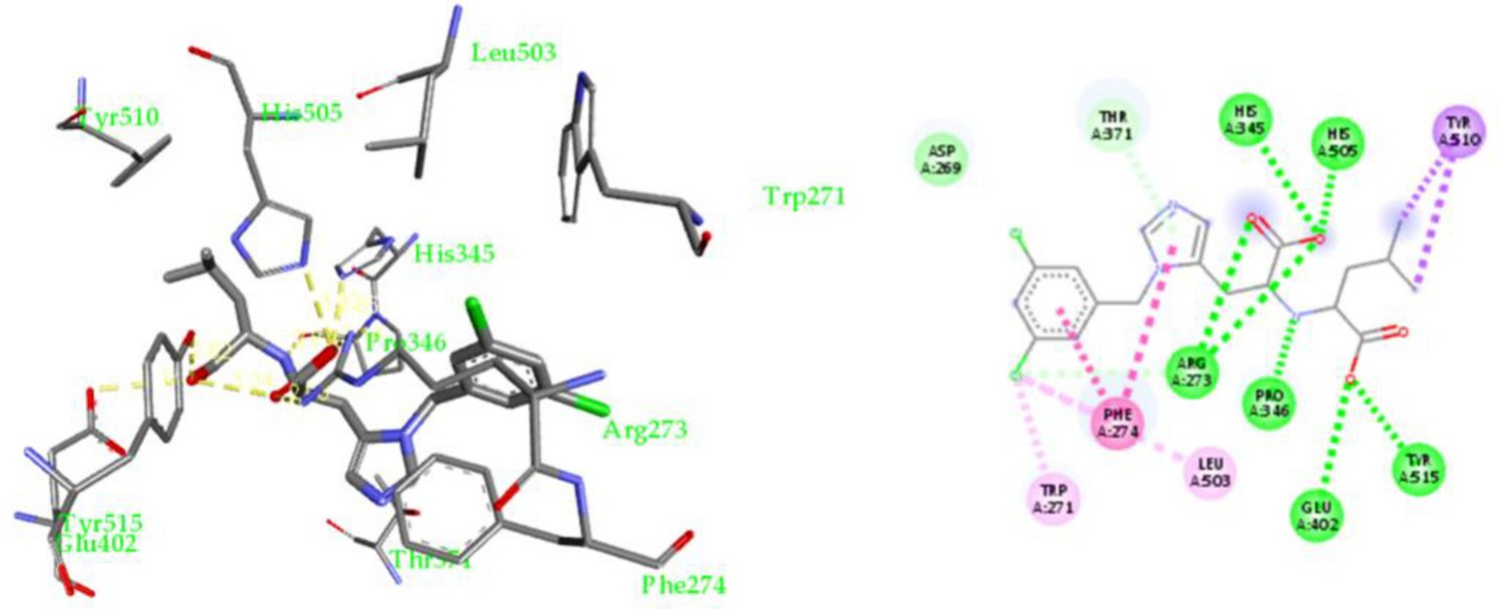
X-ray crystal structure of MLN-4760 bound to human ACE-2 active site and 2-D schematic representation for its non-covalent interactions.

We herein investigated the potential use of these compounds as SARS-Cov-2 inhibitors by targeting the interface between the ACE-2 receptor and the SARS-CoV-2 spike RBD (**S27–S32 Figs**). The solved crystal structure of the SARS-CoV-2 spike RBD complexed with ACE-2 (PDB: 6LZG) [81] was used (**Fig 2**). We used quercetin as a reference natural drug to validate the docking parameters.

**Fig 3:**
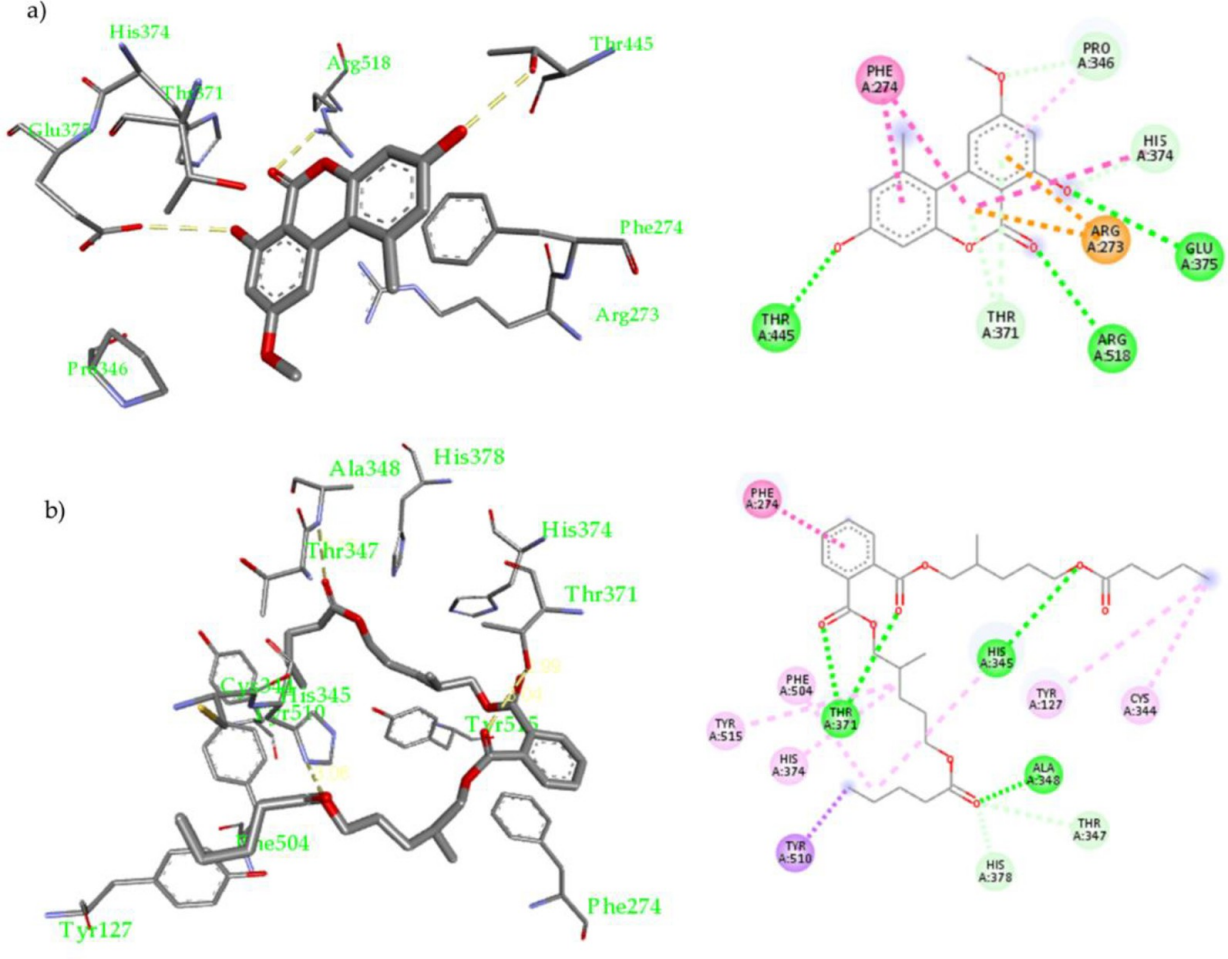
Docking of compounds **4 (a)** and **2 (b)** inside the ACE-2 active site; 3-D representation (left side) and 2-D schematic representation (right side) of their non-covalent interactions inside the ACE-2 active site.

We compared our docking results with what was previously published as we found an agreement with the literature. Quercetin was docked into the binding interface between the ACE-2 and viral (S) RBD, and it was found that nine possible conformers showed good binding affinity (-8.9 to -7.9 kcal/mol). Quercetin forms multiple non-covalent interactions, as shown in **S33 Fig** Using the validated docking parameters, molecular docking of compounds **2** and **4** was performed on the crystal structure of the SARS-CoV-2 spike RBD complexed with ACE-2 (**Fig 3**).

Compound **2** was docked into the binding interface between the ACE-2 and viral (S) RBD, and nine possible conformers resulted in having an excellent binding affinity (-7.3 to -6.3 kcal/mol). It was found that **2** fit in the ACE-2 -Spike RBD site of interaction forming numerous non-covalent interactions, **Fig 3A**: 1) H-bonds with Asn33 (3.15 Å), Phe390 (2.94 Å), Arg393 (2.88 Å and 3.08 Å), Arg403 (2.81 Å) and Tyr505 (2.97 Å); 2). Hydrophobic interactions with His34 (4.40 Å) and Val93 (4.73 Å). Compound **4** was docked using the same parameters (**Fig 3** and **S27 Fig**), and nine possible conforms resulted in a binding affinity (–6.9 to -6.1 kcal/mol). It was found that **4** fit nicely in the ACE-2-Spike RBD site of interaction forming many non-covalent interactions, (**Fig 3**) H-bonds with His34 (3.19 Å), Arg393 (2.95 Å), Glu406 (2.87 Å), Arg408 (3.04 Å), Gln409 (2.94 Å) and Tyr505 (2.96 Å); 2) Hydrophobic interactions with Ala386 and Arg403. A table with full details about the docking experiment is included in the **S2 Table**, **S28–S33 Figs**.

### Inhibitory effect of endophytic compounds 1–4 on ACE-2 / Spike (RBD) protein interaction

**Fig 4:**
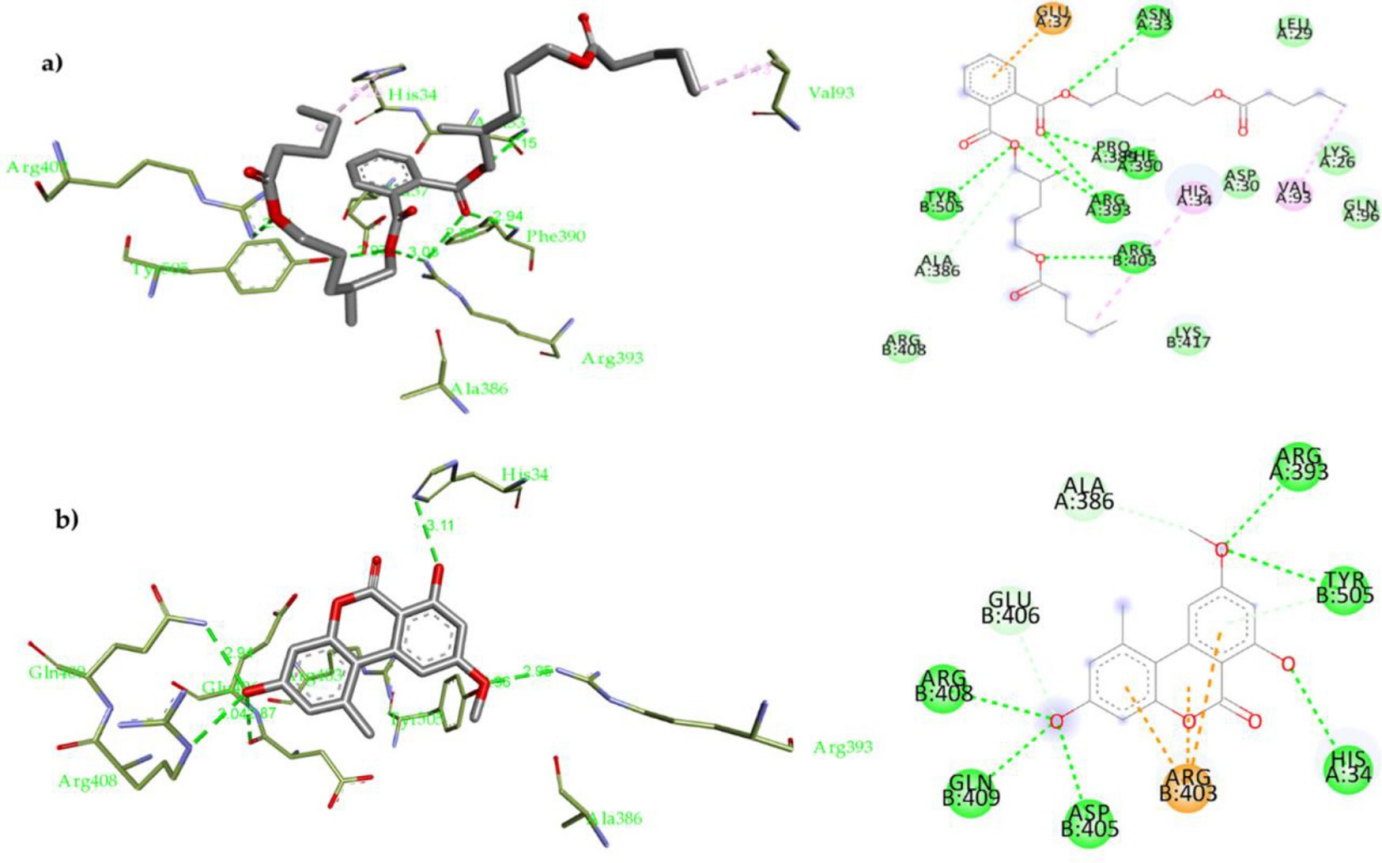
Docking of compounds **2 (a)** and 4 **(b)** inside the binding site of SARS-CoV-2 spike RDB in complex with ACE-2; 3-D representation (left side) and 2-D schematic representation (right side) of their non-covalent interactions inside the binding site of SARS-CoV-2 spike RDB in complex with ACE-2.

Results shown in **Table 3** and **S34 Fig** revealed that compounds **1–4** can efficiently inhibit the interaction between ACE-2 SARS-CoV-2 Spike (RBD) protein interaction compared to quercetin (IC_50_, 16.53 ± 0.44 μM). Compounds **2** and **4** were the most active, with IC_50_ of 10.40 ± 0.25 and 11.70 ± 0.34 μM, respectively. Moreover, EtOAc extract showed potential activity with IC_50_ of 0.60 ± 0.02 μg/mL (**Fig 4**).

**Table 3:** *In vitro* ACE2 spike RBD (SARS-CoV-2) inhibitory effect of compounds 1–4 compared to quercetin.

Compound	IC_50_ (μM)
**1**	15.50 ± 0.45
**2**	10.40 ± 0.25
**3**	12.50 ± 0.46
**4**	11.70 ± 0.34
**Quercetin**	16.53 ± 0.44

Data is presented as mean ± SD.

### Molecular dynamic simulation results

#### RMSD and RMSF analysis

**Fig 5:**
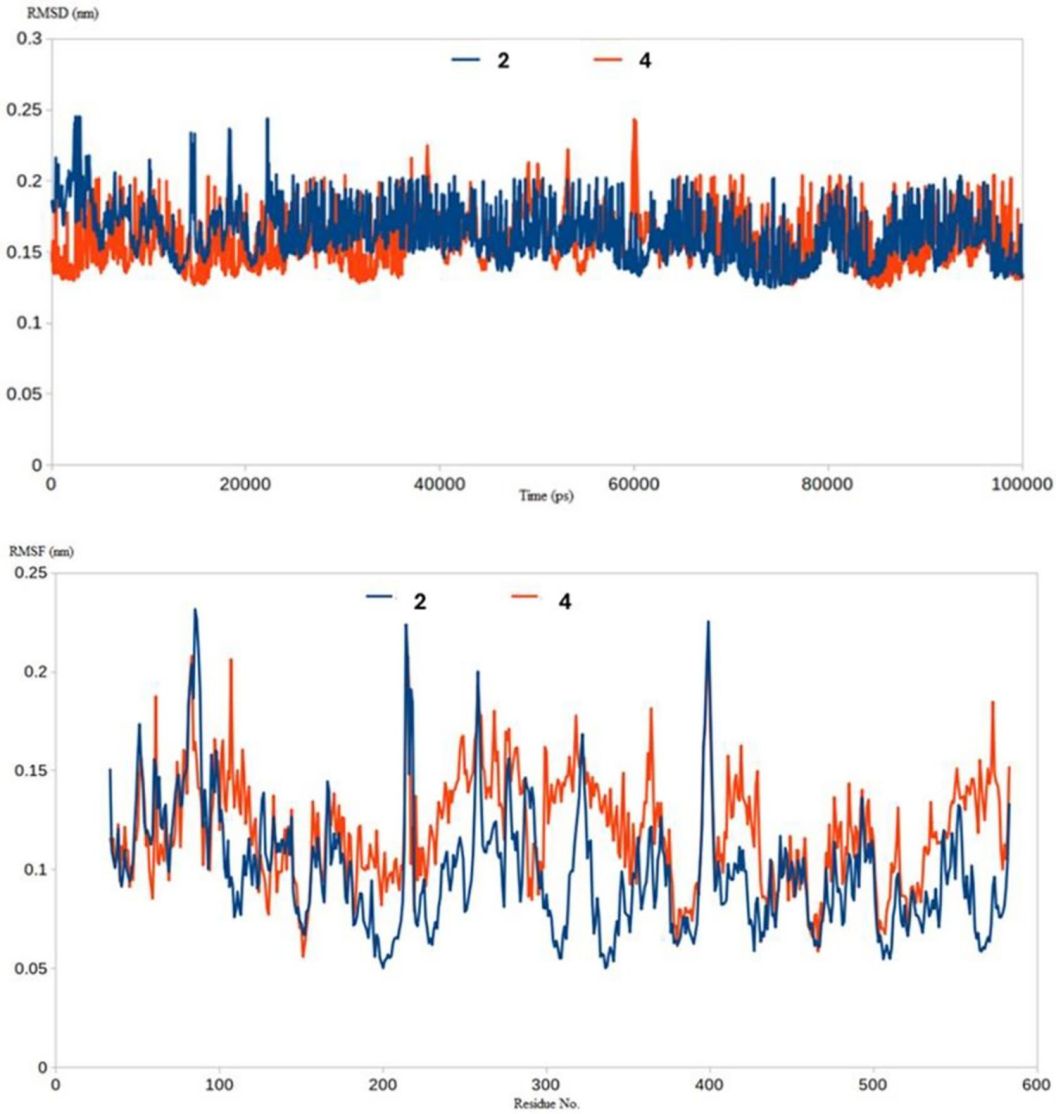
RMSD (Top) and RMSF (Bottom) analysis for the MD simulations of Compound **2** (blue) and **4** (red) inside the hACE2 active site.

Molecular dynamic (MD) simulation provides many valuable information and parameters to study the dynamicity of biological complexes. MD could provide insights into precise estimation of the binding strength of a docked complex of a small molecule and a receptor protein. In this study, we performed some initial computational investigations through molecular dynamic simulations to assess the stability of the complex between our compounds and the *h*ACE2 active site (**S35 Fig**). Compounds **2** and **4** were found to be able to form a stable complex with the active site of ACE2 protein as indicated by their lower RMSD values. Compound **2** and **4** complexes had RMSD values of 0.19 nm and 0.2 nm, respectively. Similar results were obtained from the RMSF analysis where compound **2** and **4** complexes showed acceptable stabilities with an average RMSF of 0.17 and 0.18 nm, respectively **(Fig 5)**.

The ability of compounds **2** and **4** to form stable complexes as indicated by the low RMSD and RMSF values is a valid indicator on their inhibitory effect on *h*ACE2 protein. Binding Free Energy Calculations using *MM-PBSA* approach. We followed up with binding energy calculation to better understand the strength and stability of the complex formed between our compounds and *h*ACE2 residues. This was done using the g_mmpbsa package to calculate the binding free energies (ΔE _binding_) between the two compounds and the ACE2 residues. We have used these tools to calculate all the forms of binding free energy including electrostatic, van der Waal, solvation and SASA energies (**Table 4)**. The calculated binding free energy for both compounds supported the two compounds are expected to form a stable and strong interactions with the *h*ACE2 active site residues preventing SARS-CoV-2 S protein interactions and blocking coronavirus from entering the host cells.

**Table 4:** The binding free energies (ΔE) of compound 2 and 4 in complex with *h*ACE2.

	ΔE _binding (kJ/mol)_	ΔE _Electrostatic (kJ/mol)_	ΔE _*Vander Waal* (kJ/mol)_	ΔE _solvation (kJ/mol)_	SASA _(kJ/mol)_
**2**	-87± 3.5	-56.1±2.5	-70.1± 2.9	57.0± 5.2	-13.7± 0.1
**4**	-81.9± 2.3	-54.1±1.5	-58.7± 1.8	49.4± 2.6	-15.8± 0.1

### *In-silico* pharmacokinetic results

The physicochemical properties and pharmacokinetics of Lipinski’s tested active compounds Prediction were estimated by the online tool SwissADME. Lipinski has here stated that if a compound follows the following parameters including molecular weight < 500 g/mol, hydrogen bond acceptor active sites < 10, hydrogen bond donor active sites < 5, and lipophilicity value LogP ≤5, then, the compound is recommended to be with oral bioavailability. The study showed that active compound **4** followed the rules of Lipinski, with strong oral bioavailability, permeability, and absorption (**Table 5**) (**S36 Fig**) [82].

**Table 5:** Physicochemical and pharmacokinetic properties of the active compounds.

Compounds	2	4
**Physicochemical properties**		
Formula	C_30_H_46_O_8_	C_15_H_12_O_5_
Molecular weight	534.65 g/mol	272.25
Heavy atoms	38	20
Aromatic heavy atoms	6	14
Fraction Csp3	0.67	0.13
Rotatable bonds	24	1
H-bond acceptors	8	5
H-bond donors	0	2
Molar refractivity	147.71	75.49
TPSA	105.20 A^0^	79.90 A^0^
**Lipophilicity & water solubility**		
Log *p*	6.29	2.27
ESOL Class	Poor water solubility	Moderate water solubility
**Pharmacokinetic parameters**		
GI absorption	Low	High
BBB permeant	No	No
CYP1A2 inhibitor	No	Yes
CYP2C19 inhibitor	No	No
CYP2C9 inhibitor	Yes	No
CYP2D6 inhibitor	No	No
CYP3A4 inhibitor	Yes	No
log Kp (cm/s)	-4.61	-5.68

*GI: gastrointestinal, BBB: blood brain barrier, CYP: Cytochrome P

### Inhibition of ACE-2 expression on human lung microvascular endothelial cells (HLMEC)

**Fig 6:**
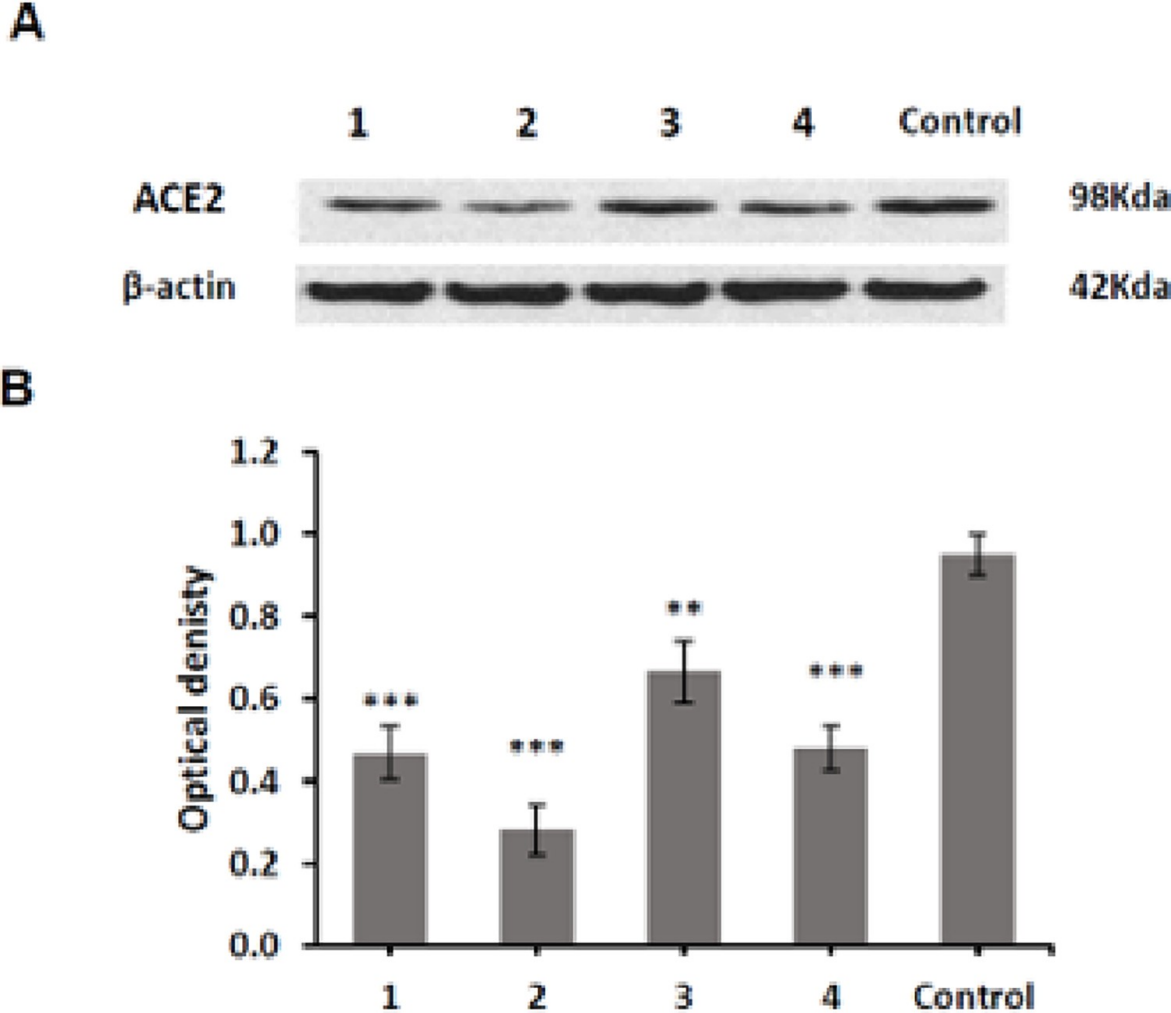
Western blot analysis of ACE-2 protein expression in human lung microvascular endothelial cells (HLMEC). **(A)** One of three repeated experiments is displayed in the above figure. **(B)** Quantitative analysis of ACE-2 protein expression using bar chart**and ***: Significantly different from negative control at P <0.01 and P <0.001, respectively using One Way ANOVA followed by post hoc test.

An experimental model of HLMEC was applied to study the effects of the endophytic compounds **1–4** on ACE-2 protein expression. Preceding this analysis, the cytotoxic effect of compounds **1–4** was determined on HLMEC using an MTT assay. HLMEC were treated with different dilutions of the test samples (0.39–100 μM) for 48 h. Compounds **1–4** being nontoxic (IC_50_>100 μM, **Table 6)**. Then, their effect on ACE-2 protein expression at the selected dose (25μM) [83] was evaluated by western blot and revealed that ACE-2 receptor protein expression was significantly inhibited by all compounds with **2** and **4** showing the greatest activity, compared to control non treated cells, (**Fig 6** and **Table 6**).

**Table 6:** Viability of HLMEC and inhibitory effects on ACE2 protein expression in HLMEC by of compounds 1–4.

Compound	IC_50_ (μM)	ACE2% inhibition
**1**	145.45 ± 4.82	49.65 ± 3.06***
**2**	154.73 ± 5.22	70.51± 4.80***
**3**	117.72 ± 6.61	29.91 ± 4.26**
**4**	151.07 ± 4.47	50.85 ± 4.34***

Values correspond to the mean ± SD of three independent experiments. p-values < 0.01** and 0.001*** are statistically significant for treated sample vs. the negative control (untreated HLMEC) using One way ANOVA followed by post hoc test.

### Anti-inflammatory activity of endophytic compounds

**Fig 7:**
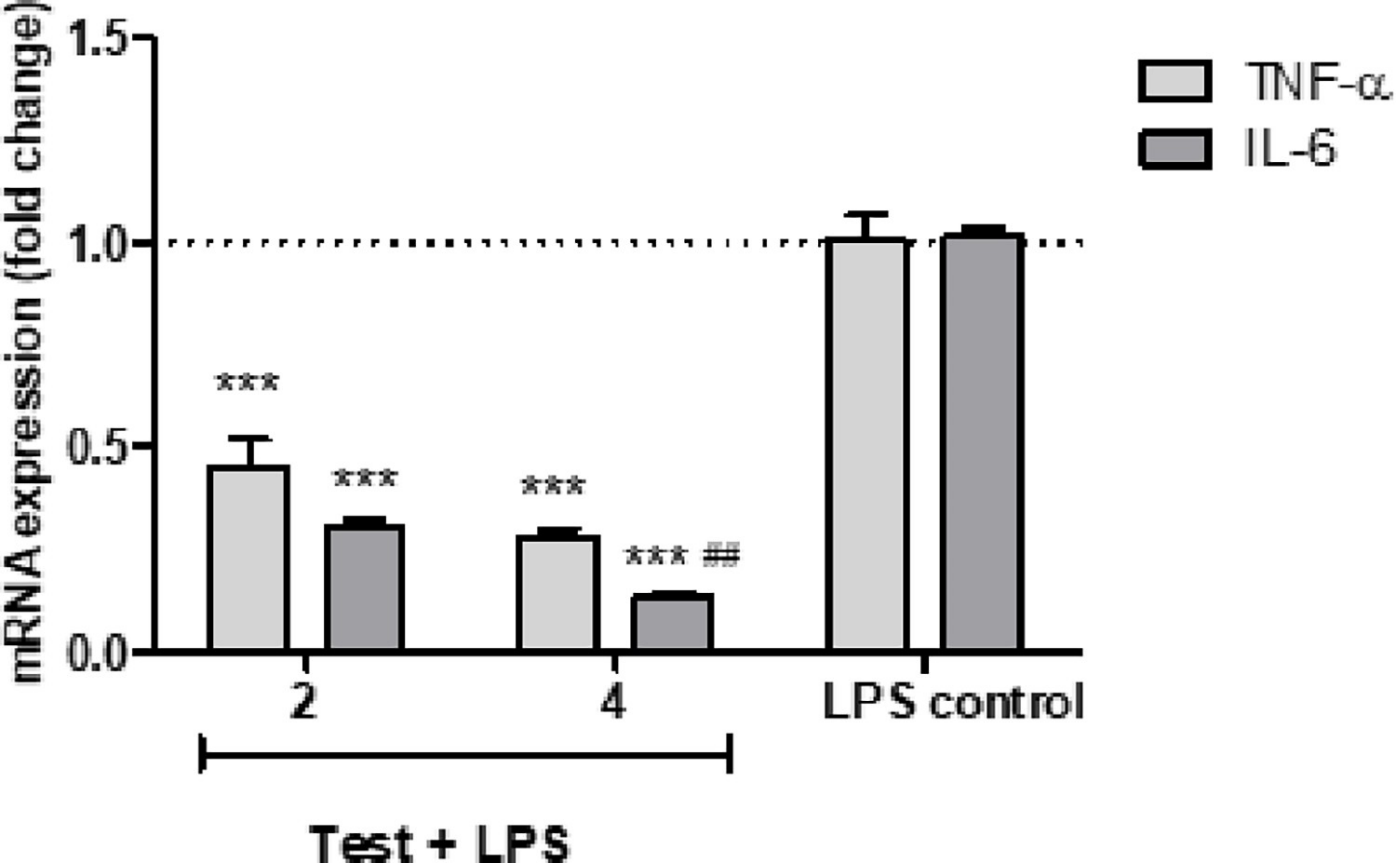
The effect of compounds 2 and 4 on TNF-α and IL-6 mRNA in LPS-stimulated RAW 264.7 macrophages. Cells were stimulated with LPS (1 μg/ mL). Values are presented as mean ± SD mRNA expression fold changes of three tests referenced to Actb: the housekeeping gene. ***, ##: Significant at p < 0.001 and < 0.01 compared to the mRNA levels of the LPS control group and compound 2, respectively.

All tested compounds (**Table 7** and **S37 Fig)** showed significant TNF-α: TNFR2 inhibitory activity while compounds **2** and **4** demonstrated the most active inhibitory effect compared to the positive control, certolizumab. Also, compound **3** showed the same activity as certolizumab. EtOAc extract inhibited the TNF-α: TNFR2 with IC_50_, 0.76 ± 0.04 μg/mL. Additionally, the alteration in TNF-α and IL-6 levels following the stimulation of RAW264.7 cells by LPS as a cell-based anti-inflammatory assay was estimated using quantitative real-time PCR. We noticed that both compounds exerted significant (p < 0.001) anti-inflammatory effects as indicated by the diminished expression of TNF-α and IL-6 mRNA. Compound **4** demonstrated higher anti-inflammatory activity than **2** as indicated by the downregulation of the TNF-α and IL-6 genes expression (fold change; 0.28 ± 0.02 and 0.14 ± 0.01, respectively versus 0.45 ± 0.07 and 0.30 ± 0.02, respectively) compared to their corresponding levels in the LPS control, (**Fig 7, S37 Fig)**.

**Table 7:** *In vitro* TNF-α inhibitory effect of the compounds 1–4.

Compound	IC_50_ (μM)
**1**	8.70 ± 0.34
**2**	5.40 ± 0.33
**3**	6.70 ± 0.39
**4**	4.97± 0.28
**Certolizumab**	6.70 ± 0.34

Data is presented as mean ± SD.

## Discussion

Several studies concluded that the ACE-2 receptor is the entry gate SARS-CoV-2 uses to enter target cells [84, 85]. SARS-CoV-2 attaches to human ACE-2 receptor *via* the binding of spike (S) glycoprotein and uses it to bind to the host’s cell surface and promote the virus-cell membrane fusion into the host cell through the viral infection [86]. The SARS-CoV-2 S protein is a homotrimer class I fusion protein in a metastable conformation [87]. The S1 subunit comprises the RBD responsible for attaching to the host ACE-2 receptor and contributes to the stability of the S2 subunit state before the fusion process. It was found that the engagement of ACE-2 with the spike RBD involves conformational changes that intermediary expose (open state) or hide (closed state) other (S) protein binding domains required for the receptor binding [87]. The open state is the state that triggers S2 subunit fusion and viral infection. Several recently solved crystal structures for SARS-CoV-2 spike receptor binding domain complexed to ACE-2 revealed that the most critical ACE-2 residues that straight interact with SARS-CoV-2 S protein RBD are: Glu 22, Glu 23, Lys 26, Asp 30, Glu 35, Glu 56, and Glu 57.

Moreover, Lys 26, Asp 30, and His 34 residues play a severe role in the interface of SARS-CoV-2 RBS S-protein to ACE-2 thus, they are essential for the entry method [8, 81, 88]. Therapeutic approaches for blocking coronavirus from entering the host cells *via* targeting Spike proteins or ACE-2 receptors are valuable for improving potential treatment for the SARS-CoV-2 viral infection [89]. In the current study, we accomplished a thorough *in-silico* analysis for evaluating the isolated compounds against the SARS-CoV-2 spike receptor binding domain complexed with *h* ACE-2. The outcome of each *in silico* evaluation is expected to rationally explain the biological evaluation data for these compounds as potential SARS-CoV-2 inhibitors. We expected that by disrupting the interactions between the SARS-CoV-2 Spike RBD and the ACE-2 residues, the entry process of the virus would be halted, and the fusion process would not occur. A previous study showed that targeting the ACE-2 amino acid residues Lys26, Asp30, and His34 residues and the RBD motif residues Arg426 and Tyr436 lead to inhibition of SARS-CoV-2 S protein from interacting with *h*ACE-2 [90]. The results of the *in-silico* analysis for all compounds binding showed that these compounds bind nicely to the interacting site between the ACE-2 and the SARS-CoV-2 S protein RBD binding, proposing that these metabolites can be used as potential SARS-CoV-2 inhibitors. For additional analysis, the biological evaluation of these compounds was performed.

Because of the severe sequels of the COVID-19 infection epidemic, discovering a probable means for inhibition of viral virulence and invasion is of ultimate value [91]. Thus, in the current research study, guided by the molecular docking results, we assessed the two new compounds (**1** and **2**) together with three known compounds (**3–5**) [33, 79, 80] isolated from the EtOAc extract of A. alternate for their ability to hinder the interaction between ACE-2 and SARS-CoV-2 spike-protein receptor binding domains. We found that agreeing with the molecular docking results, those compounds may offer promising protection against viral infection by blocking this entry path for SARS-CoV-2 with IC_50_ values lower than 16.53 ± 0.44 μM, which corresponds to that of quercetin; the most efficient ACE-2 fixative among other polyphenolic compounds [92] (S29 and S30 Figs).

An additional novel part of our study included experimental proof of the effectiveness of the endophytic metabolites in hindering ACE-2 receptor expression in the microvascular lung endothelial cells. We found that compounds **2** (% inhibition, 70.51± 4.80) and **4** (% inhibition, 50.85 ± 4.34) demonstrated the most potent inhibitory activity (P<0.001) against ACE-2 receptor expression. Several metabolites obtained from microbial fungi and bacteria similar to the chemical class of our compounds were studied for their inhibition of ACE-2 with SARS-CoV-2 [93]. Moreover, the previous docking and dynamic study for compounds **4** and **5** and other microbial compounds revealed their ability to inhibit SARS-CoV-2 [94]. During the life cycle of the virus, several factors can control the host’s response toward certain viral infections [95]. The initial disease stage includes the viral phase with the appearance of some symptoms. As the infection proceeds, the inflammatory phase substitutes the viral phase to manage the viral replication. However, the host cells are damaged [96]. Therefore, antiviral drugs are active during the viral phase and then become ineffective [97], while treatment options for managing inflammatory infections during the inflammatory phase, include using anti-inflammatory agents [26].

Lately, increasing studies point out that the "cytokine storm," characterized by the intense release of TNF-α and IL-6, could contribute to the mortality caused by COVID-19 [98]. The interaction between ACE-2 and SARS-CoV-2 is a vital issue in viral infections, causing the release of inflammatory proteins like TNF-α [99]. This pleiotropic pro-inflammatory cytokine binds to a membrane receptor called tumor necrosis factor receptor 2 (TNFR2), mainly expressed in the immune system cells, like the regulatory T cells. The excessive release of TNF-α, as a part of the cytokine storm, provokes vascular and capillary barrier dysfunction, diffusing alveolar damage, and multi-organ failure. Likewise, it plays a severe role in disturbing the lung epithelial and endothelial barriers, which may have been revealed to ARDS [100]. Owing to this critical role of TNF-α in prompting COVID-19-related cytokine storm syndrome, it is crucial to screen for new approaches as anti-TNF therapy [101].

Due to the close relation between ACE-2 inhibition and the anti-TNF-α agents and encouraged by the promising ACE-2 inhibition showed by the endophytic compounds thus, in the present work, we checked the impact of secondary metabolites and the EtOAc extract on TNF-α inhibition, initially by an *in vitro* assay which showed that compounds **2** and **4** exhibited the most effective inhibition with IC_50_ values lower than 6.70 ± 0.34 μM, which corresponds to certolizumab.

LPS-stimulated macrophages produce undue levels of cytokines such as TNF-α and IL-6 [102]. Thus, we performed an *in vitro* cell-based anti-inflammatory effect for the most active metabolites *viz* **2** and **4** using LPS-induced RAW 264.7 cells to assess their role against the cytokine response by estimating TNF-α and IL-6 levels. Both compounds revealed a potential anti-inflammatory activity, however, compound **4** was more effective causing significant downregulation (P<0.001) of both genes. Likewise, it was reported that the extracts of *A*. *alternate* exhibited reduced TNF-α production in LPS-stimulated macrophages [103, 104]. Hence, the current study revealed the bioactive potentiality of the tested compounds from the EtOAc extract of *A*. *alternate* isolated from the leaves of *C*. *viminalis* against SARS-CoV-2. Additionally, the promising anti-inflammatory activities shown by these compounds can be beneficial for the treatment of several inflammatory disorders such as inflammatory bowel disease, lung conditions such as ARDS, chronic obstructive pulmonary disease (COPD), or pulmonary fibrosis, diabetic nephropathy, and chronic kidney disease, in addition to neuroinflammation, neurodegeneration, and neurological disorders like Alzheimer’s disease. Since one-third of FDA-approved drugs in the US markets are natural products [105], our work focuses on the use of natural products for the management of coronavirus-related illnesses and demonstrates the promising prospects of natural products from *A*. *alternate* against coronavirus by listing some natural pure compounds isolated from *A*. *alternate* and their therapeutic means, offering references for subsequent related research studies.

Large-scale production of these biomolecules using different biotechnological tools could enhance *in vivo* and clinical studies.

## Conclusion

The assessments of microbial metabolites for antiviral activity attract researchers due to their advantages over plant, animal, and synthetic sources. Therefore, the request for antiviral microbial compounds is gradually increasing due to plant extraction and chemical synthesis need time and economic issues. Consequently, we isolated and purified *A*. *alternate* from the leaves of *C*. *viminalis*. We succeeded in isolating two novel compounds (altenuline **1**, phthalic acid bis (7’/7’’ pentyloxy) isohexyl ester **2**) along with three known ones (1-deoxyrubralactone **3,** alternariol-5-*O*-methyl ether **4** and alternariol **5**) from its EtOAc extract. Based on the *in-silico* and *in vitro* studies, all isolated compounds, especially compounds **2** and **4,** can inhibit the virus entry into the host cells by blocking the interaction between ACE-2 with CoV-19 spike-protein receptor binding domains as well, their ability to decrease ACE-2 expression which is abundantly present in human lung microvascular endothelial cells. Moreover, they exhibited promising anti-inflammatory effects through TNF-α: TNFR2 inhibitory activity in addition to their inhibitory effect on TNF-α and IL-6, the predominant cytokines in COVID-19-related cytokine storm *via* cell-based anti-inflammatory assay. The most active compounds against ACE-2 and proinflammatory cytokines were compounds **2** and **4** and the SwissADME prediction tool displayed their drug-like characters. Combining the anti- ACE-2 and anti-inflammatory activities of these compounds enhanced their predicted effectiveness to control viral invasion, and to ameliorate the disease progression. Thus, the active compounds isolated from *A*. *alternate* could be utilized as a potential ingredient in phytopharmaceuticals for managing COVID-19. However, additional studies are essential for their safety in clinical settings.

### Limitations and for future work

- Liquid culture media for isolation of the metabolites not used in this study for isolation of the compounds due to the facility’s limitations in our lab.
- To add value to our work, we may test the compounds against coronavirus using a pseudovirus entry inhibition assay followed by an infectious prototypic SARSCoV2 cytotoxic effect inhibition assay in Vero E6 cells. However, these assays were unavailable when the research study was done.
- *in vivo* study for the metabolites and the extract should be done to study their effects using animal models and explain their safety
- Clinical study should be done for the most effective compounds

## Supporting information

S1 Fig ^1^HNMR spectrum of compound 1 (400 MHz, CDCl_3_). (JPG)

S2 Fig ^13^CNMR spectrum of compound 1 (100 MHz, CDCl_3_). (JPG)

S3 Fig APT spectrum of compound 1 (100 MHz, CDCl_3_). (JPG)

S4 Fig HMQC spectrum of compound 1 (400 MHz, CDCl_3_). (JPG)

S5 Fig HMBC spectrum of compound 1 (400 MHz, CDCl_3_). (JPG)

S6 Fig H-HCOSY spectrum of compound 1 (400 MHz, CDCl_3_). (JPG)

S7 Fig Positive ESI/MS spectrum of compound 1. (JPG)

S8 Fig ^1^HNMR spectrum of compound 2 (400 MHz, CDCl_3_). (JPG)

S9 Fig ^13^CNMR spectrum of compound 2 (100 MHz, CDCl_3_). (JPG)

S10 Fig APT spectrum of compound 2 (100 MHz, CDCl_3_). (JPG)

S11 Fig HMQC spectrum expansion of compound 2 (400 MHz, CDCl_3_). (JPG)

S12 Fig HMBC spectrum of compound 2 (400 MHz, CDCl_3_). (JPG)

S13 Fig H-HCOSY spectrum of compound 2 (400 MHz, CDCl_3_). (JPG)

S14 Fig Negative ESI/MS spectrum of compound 2. (JPG)

S15 Fig ^1^HNMR spectrum of compound 3 (400 MHz, CDCl_3_). (JPG)

S16 Fig ^13^CNMR spectrum of compound3 (100 MHz, CDCl_3_). (JPG)

S17 Fig HMQC spectrum of compound 3 (400 MHz, CDCl_3_). (JPG)

S18 Fig HMBC spectrum of compound 3 (400 MHz, CDCl_3_). (JPG)

S19 Fig H-HCOSY spectrum of compound 3 (400 MHz, CDCl_3_). (JPG)

S20 Fig Negative ESI/MS spectrum of compound 3. (JPG)

S21 Fig Negative ESI/MS spectrum of compound 3. (JPG)

S22 Fig Negative ESI/MS spectrum of compound 4. (JPG)

S23 Fig Positive ESI/MS spectrum of compound 4. (JPG)

S24 Fig ^1^HNMR spectrum of compound 5. (JPG)

S25 Fig Negative ESI/MS spectrum of compound 5. (JPG)

S26 Fig Phylogenetic trees showing relationship of strain *Alternaria alternate* isolate with other related fungal species retrieved from GenBank based on their sequence homologies of 18srDNA. (JPG)

S27 Fig Docking results for compound MLN-4760 & compound 1–4 inside human ACE2 active site. (JPG)

S28 Fig 3D docked model of compound 2 &4 bound to the ACE2 protein. (JPG)

S29 Fig 3D docked model of compound 3 inside the ACE2 active site showing possible non-covalent interactions with protein residues. (JPG)

S30 Fig The inhibition of ACE2 will prevent the SARS-CoV-2 spike protein from binding [adapted from reference [86]] and as a result no viral entry to the cell. (JPG)

S31 Fig Docking results for compound 2&4 inside the binding site of SARS-Cov-2 S protein RBD in complex with ACE2. (JPG)

S32 Fig Docking of compound 2&4 inside the binding site of SARS-Cov-2 S protein RBD in complex with ACE2. (JPG)

S33 Fig Docking of quercetin inside the binding site of SARS-Cov-2 S protein RBD in complex with ACE2 (S29E Fig. Docking of Quercetin inside the binding site of SARS-Cov-2 S protein RBD in complex with ACE2. (**Quercetin** was docked into the binding interface between the ACE2 and viral (S) RBD and 9 possible conformers were obtained with good binding affinity (ranging from -8.9 to -7.9 kcal/mol)).
(JPG)

S34 Fig % inhibition of compounds 1–4 on ACE2: Spike RBD (SARS-CoV-2) interaction. Values represent % ACE2 inhibition (mean ± SD) of three replicates.
(JPG)

S35 Fig SwissADME BOILED-Egg plot comparing compounds (1–5) *in silico* ADME and drug likeness properties. (JPG)

S36 Fig The physicochemical descriptors, ADME parameters, pharmacokinetic (PK) properties and drug likeness of compounds (1–5) in this study predicted by SwissADME tool (http://www.swissadme.ch/index.php). (JPG)

S37 Fig Effects of compounds 1–4 on TNF-α inhibition compared to certolizumab. Values represent % TNF-α inhibition (mean ± SD) of three replicates.
(JPG)

S1 Table *TNF-α*, *IL-6* and *β-actin* primer sequences for RT-PCR. (DOCX)

S2 Table In silico study, describe briefly the binding affinity, binding amino acids, bond length. (DOCX)

S1 Raw images (PDF)
