# Supplementary figures and images for: Secondary metabolites of Alternaria alternate appraisal of their SARS-CoV-2 inhibitory and anti-inflammatory potentials
Source: PLoS One. 2025 Jan 24;20(1):e0313616. doi: 10.1371/journal.pone.0313616 (PMC11760621; doi:10.1371/journal.pone.0313616)

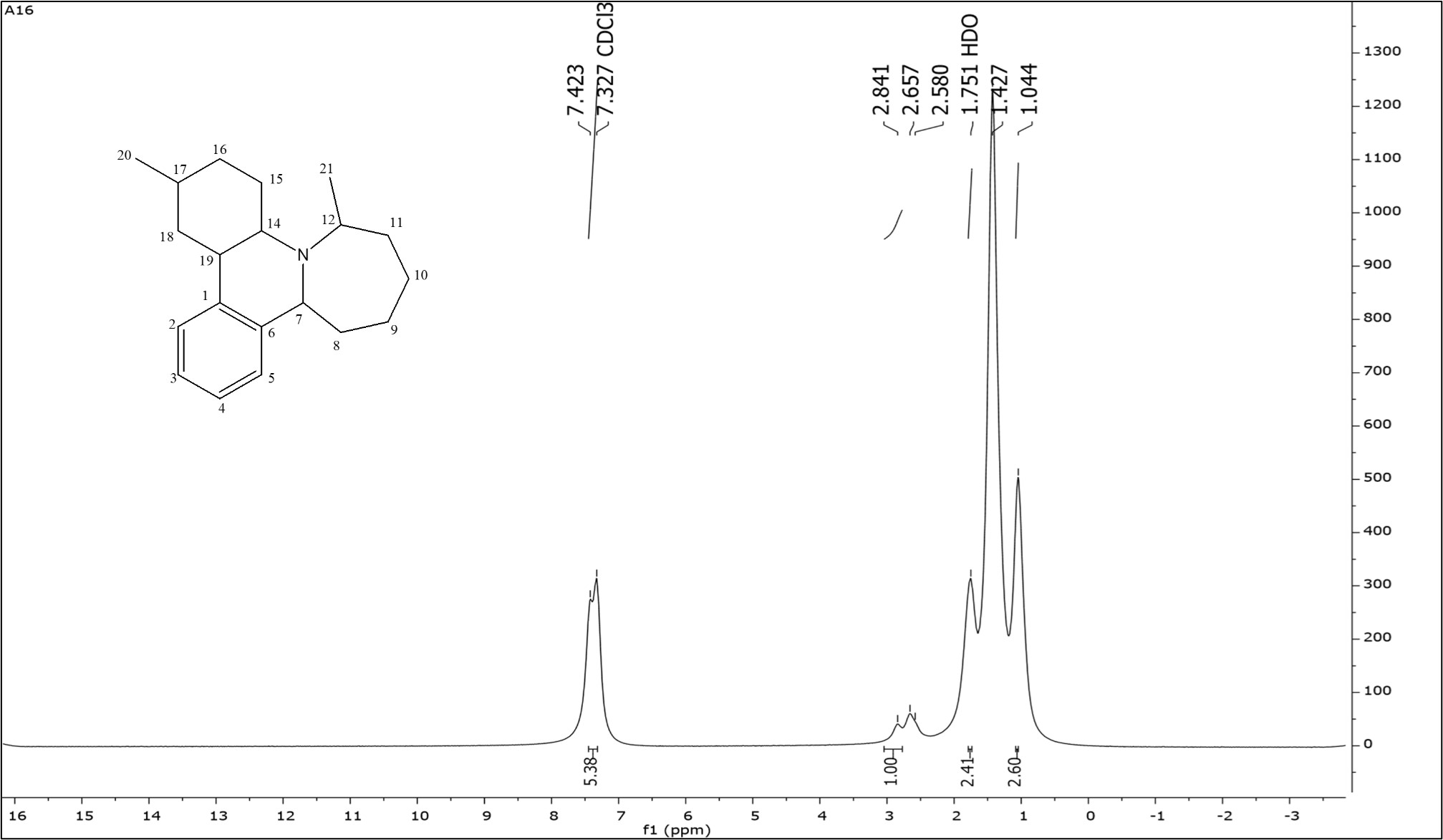

Supplement: S1 Fig — (JPG) [file pone.0313616.s001.jpg]

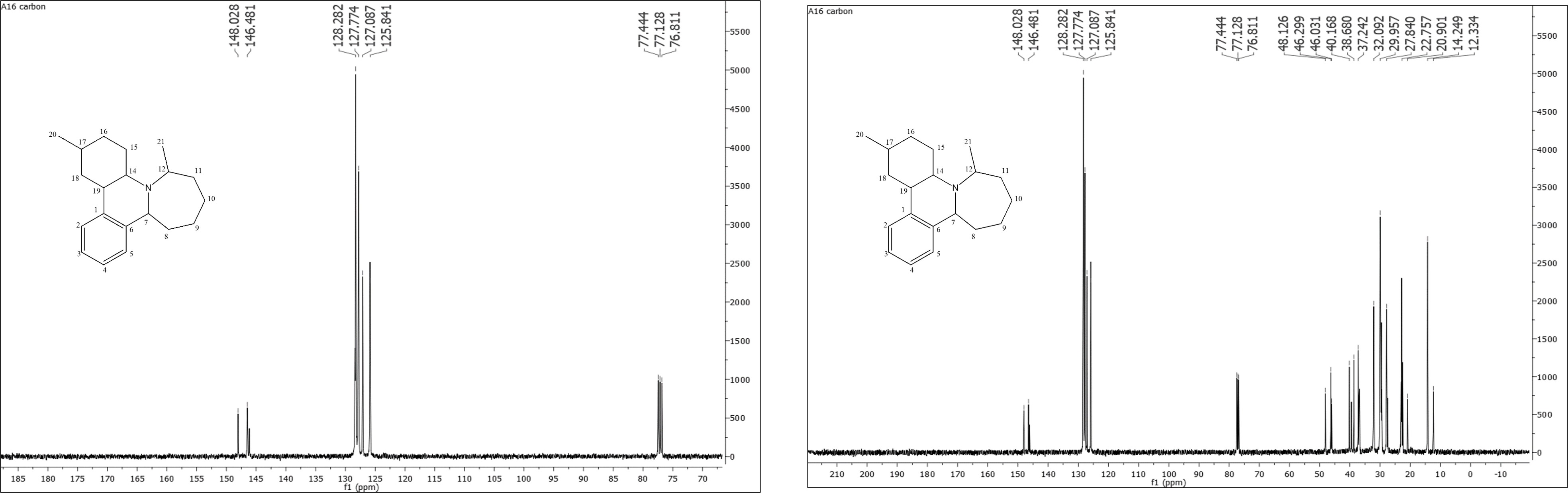

Supplement: S2 Fig — (JPG) [file pone.0313616.s002.jpg]

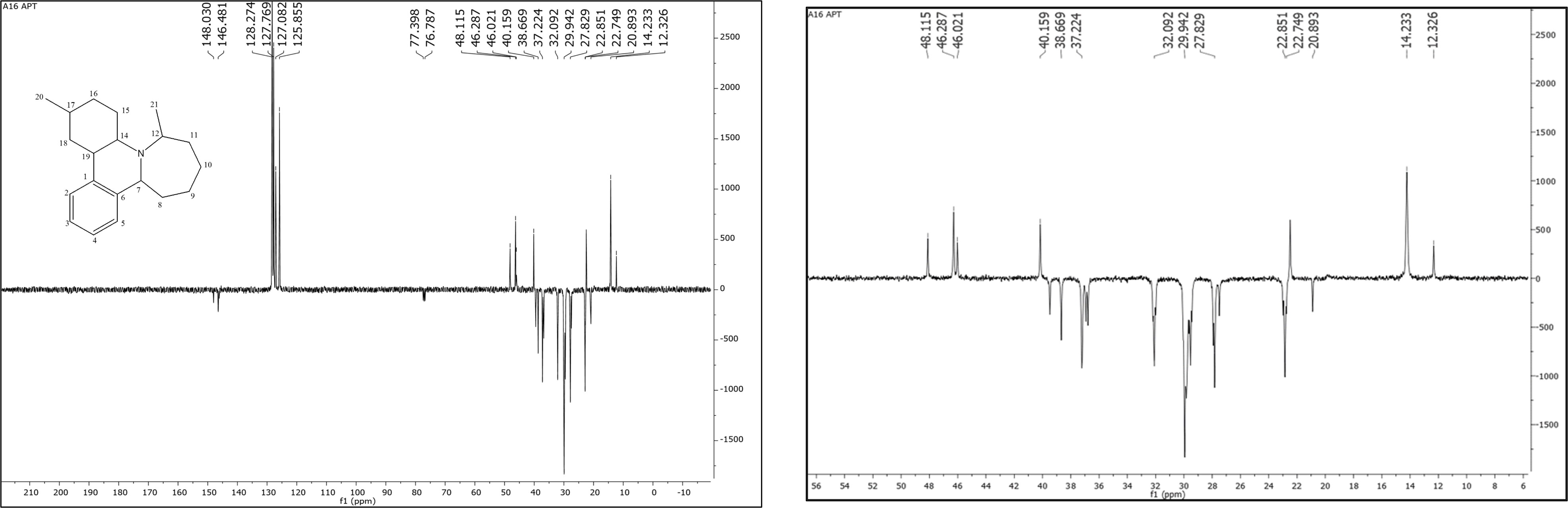

Supplement: S3 Fig — (JPG) [file pone.0313616.s003.jpg]

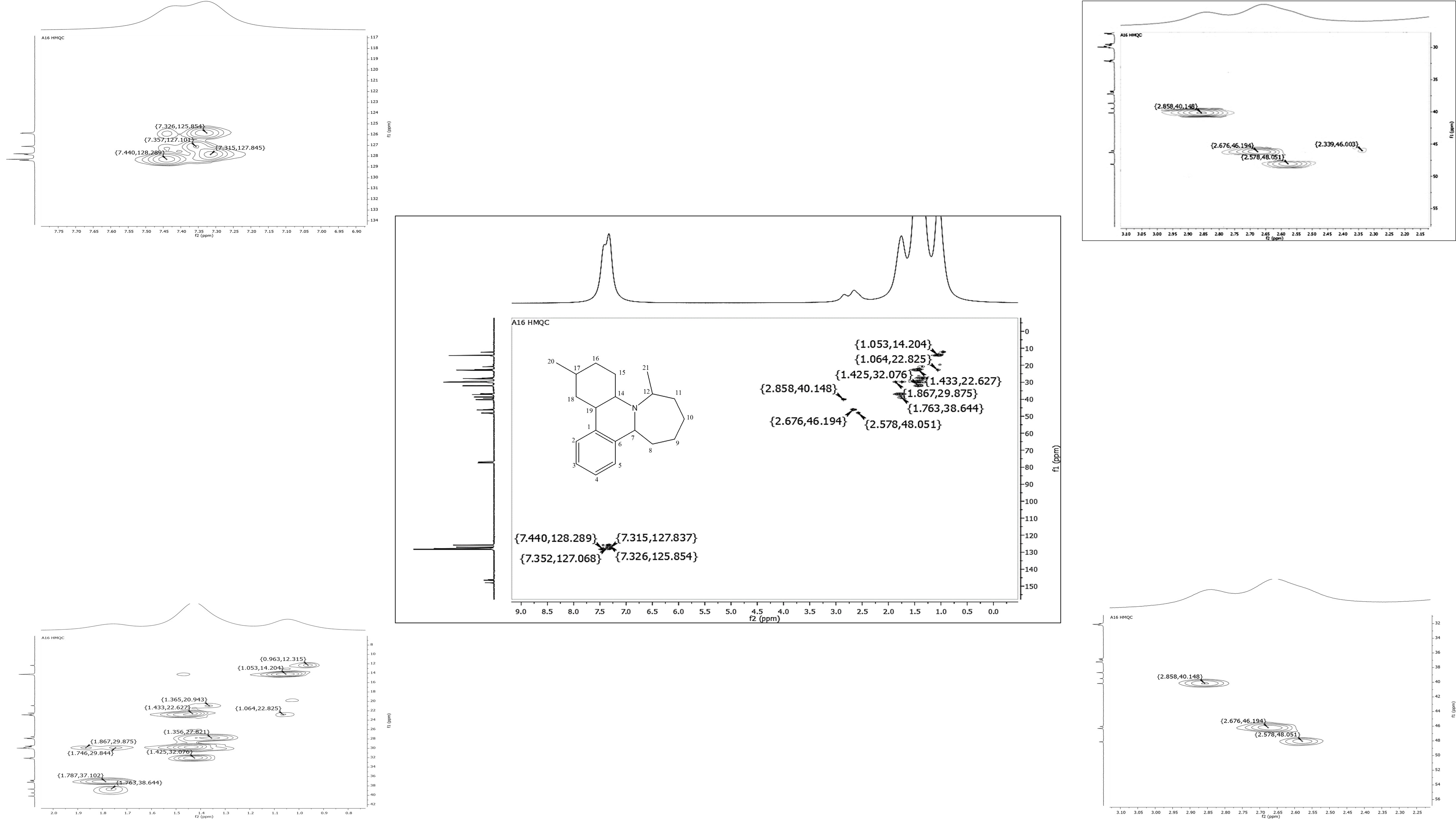

Supplement: S4 Fig — (JPG) [file pone.0313616.s004.jpg]

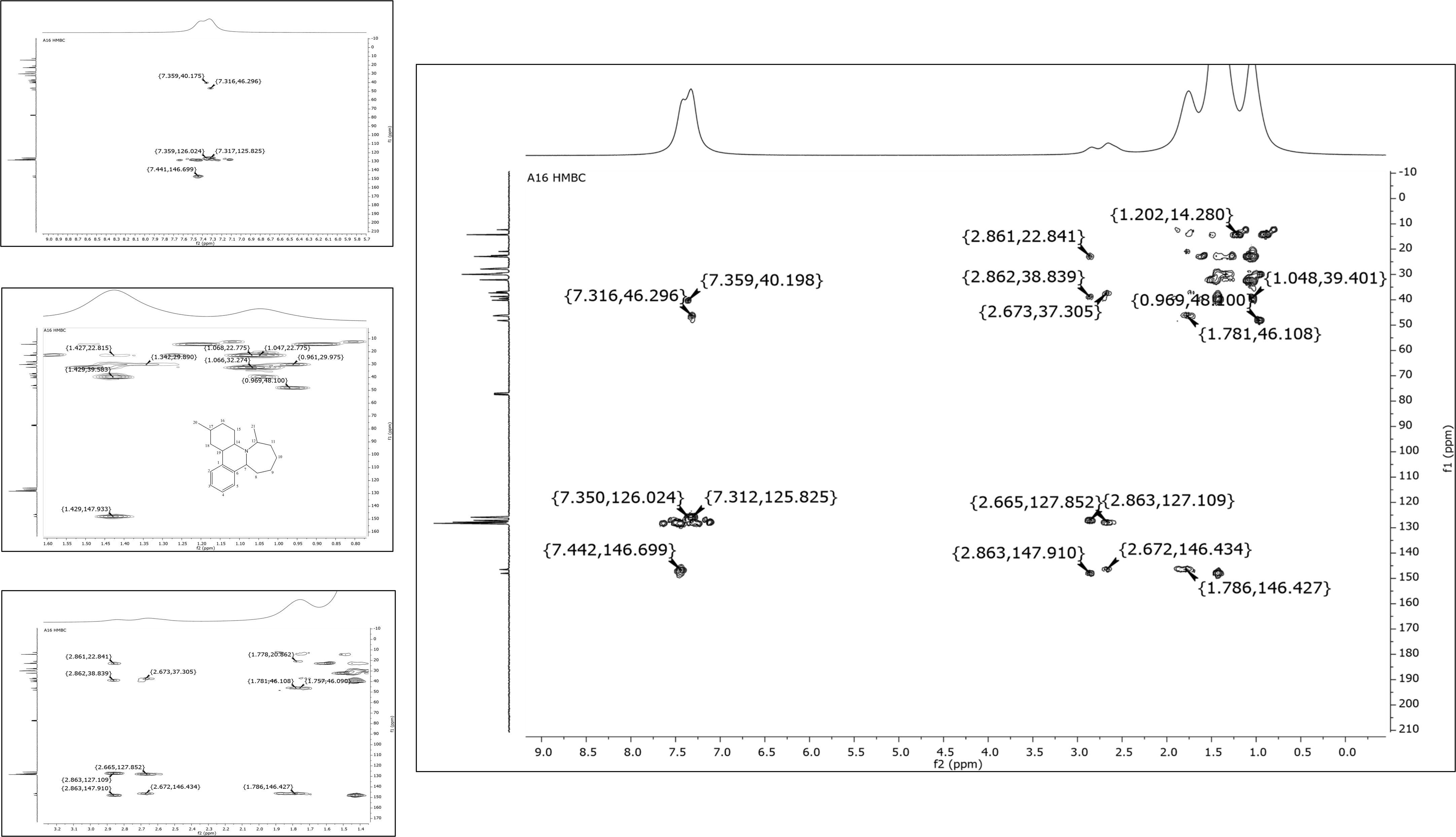

Supplement: S5 Fig — (JPG) [file pone.0313616.s005.jpg]

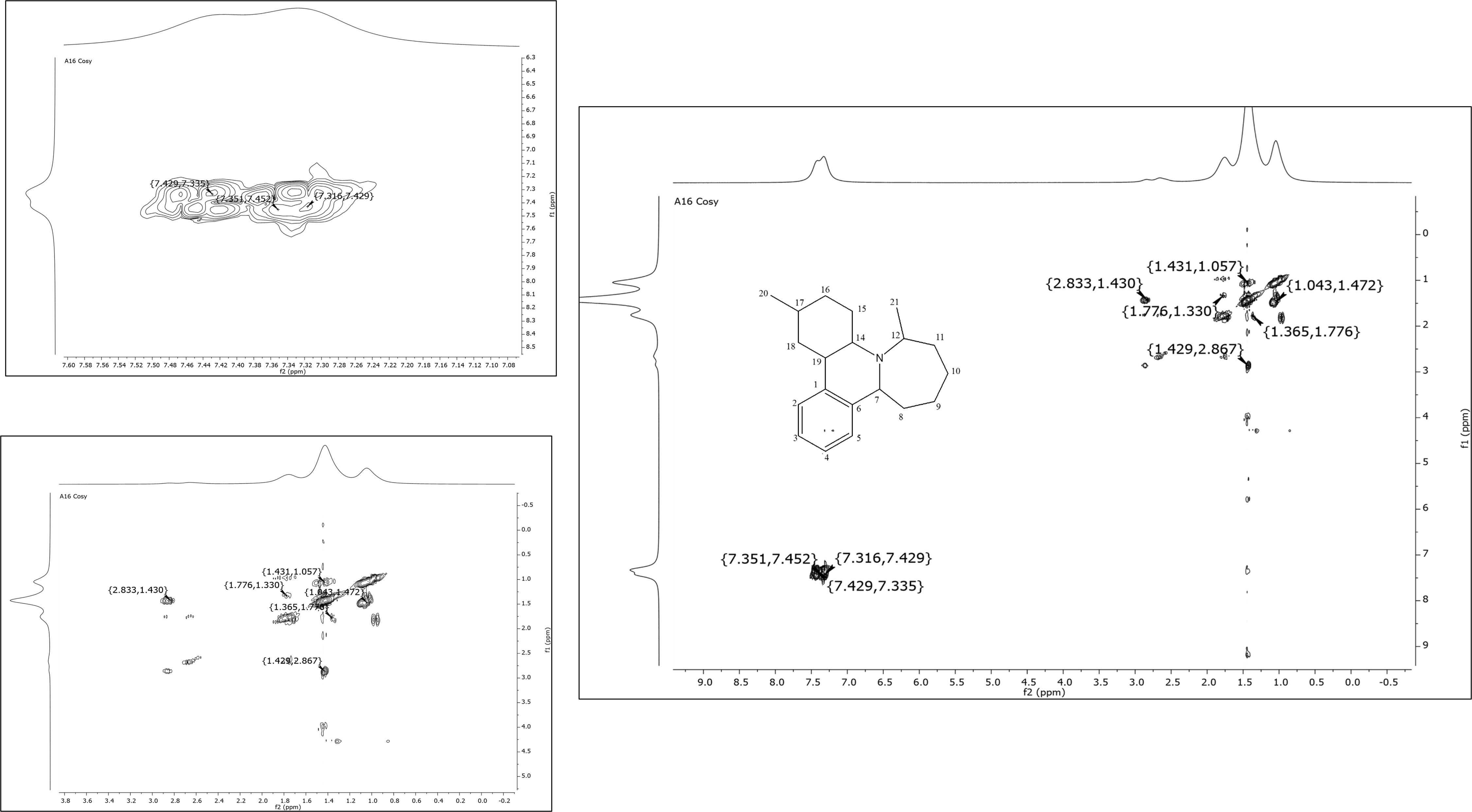

Supplement: S6 Fig — (JPG) [file pone.0313616.s006.jpg]

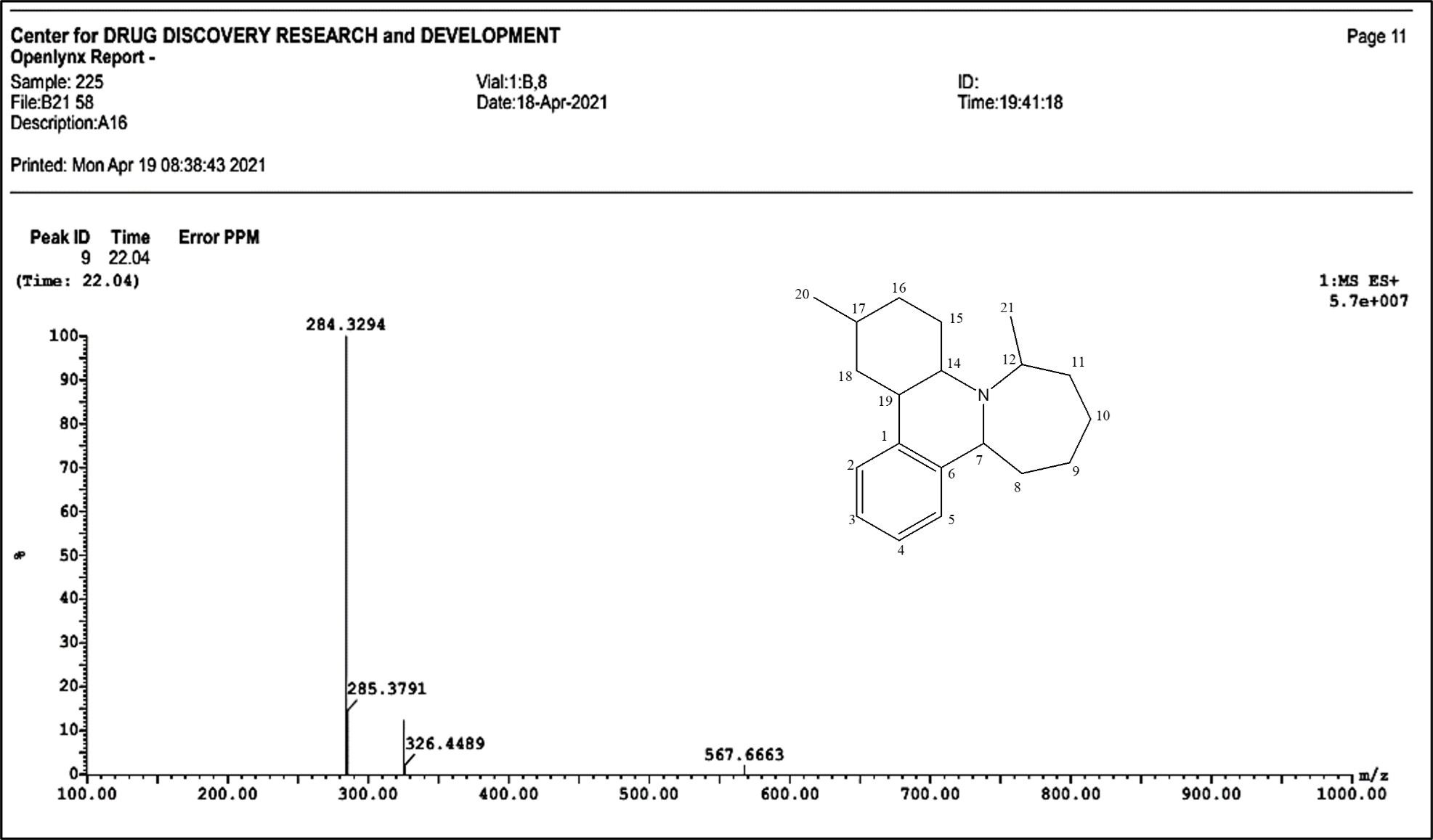

Supplement: S7 Fig — (JPG) [file pone.0313616.s007.jpg]

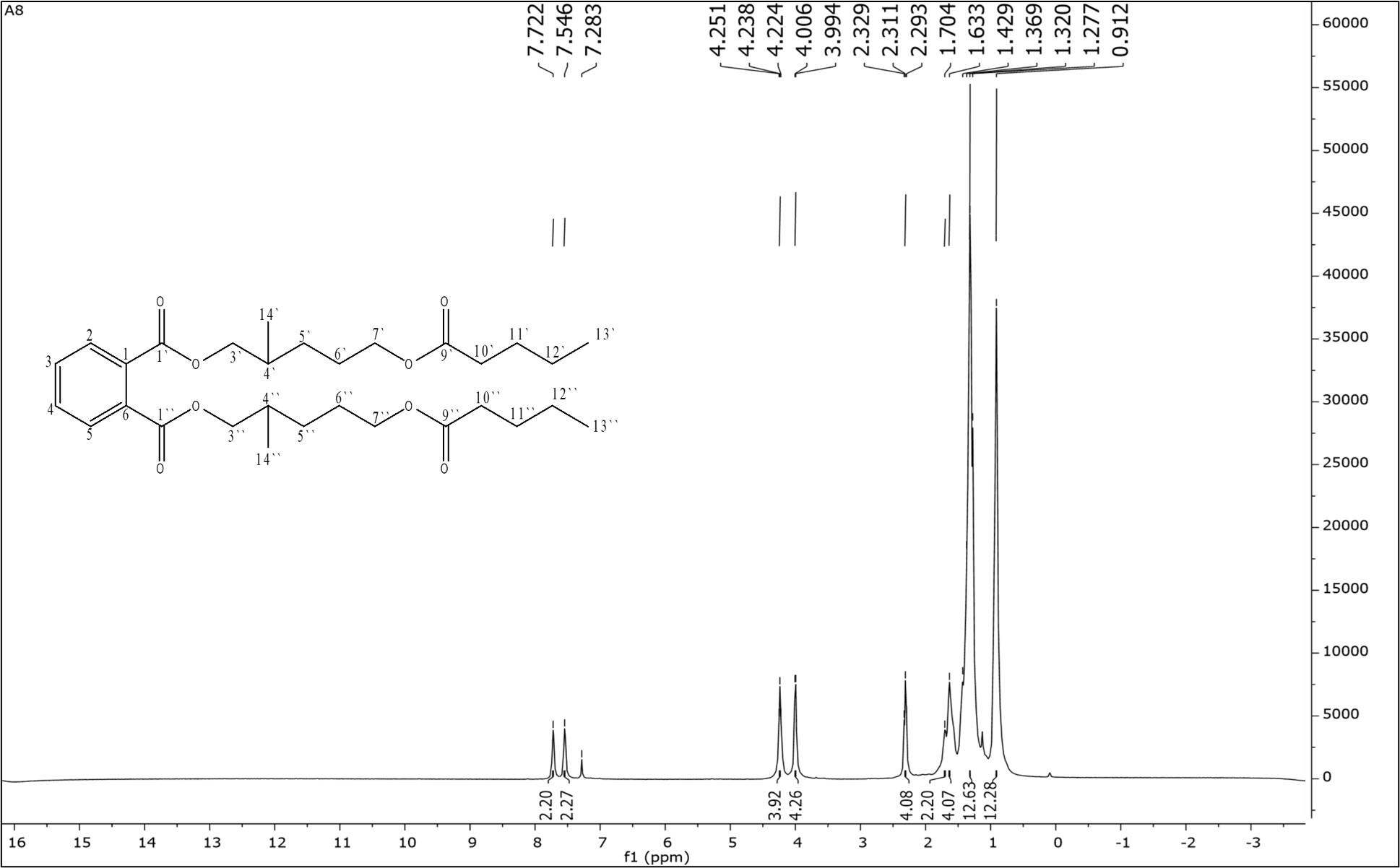

Supplement: S8 Fig — (JPG) [file pone.0313616.s008.jpg]

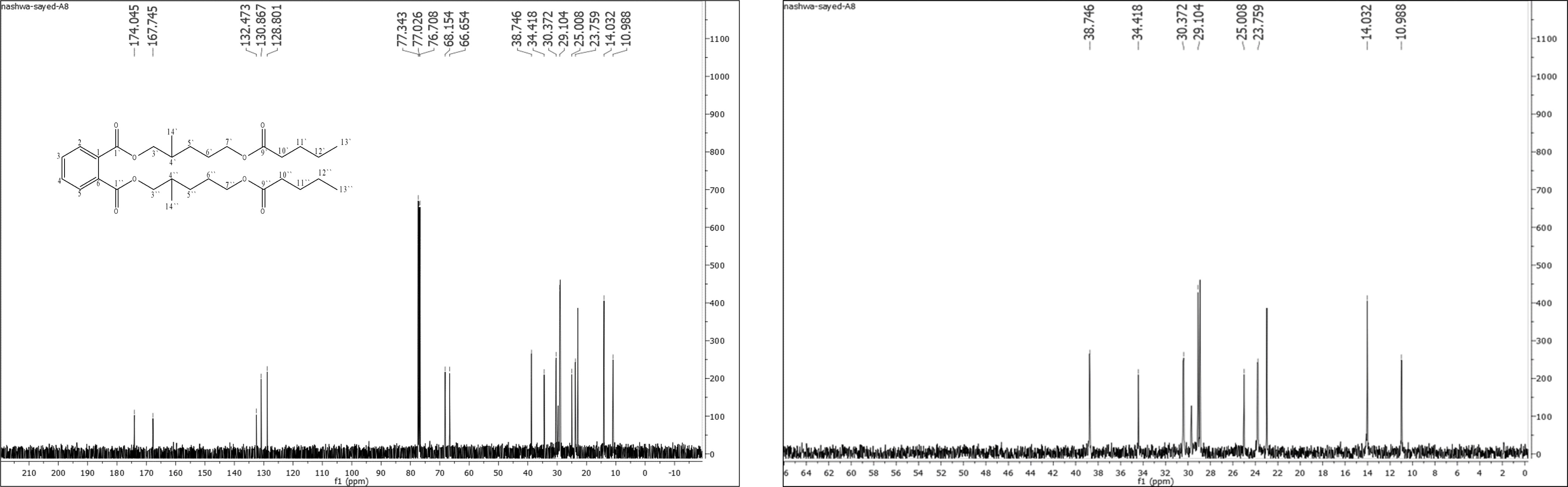

Supplement: S9 Fig — (JPG) [file pone.0313616.s009.jpg]

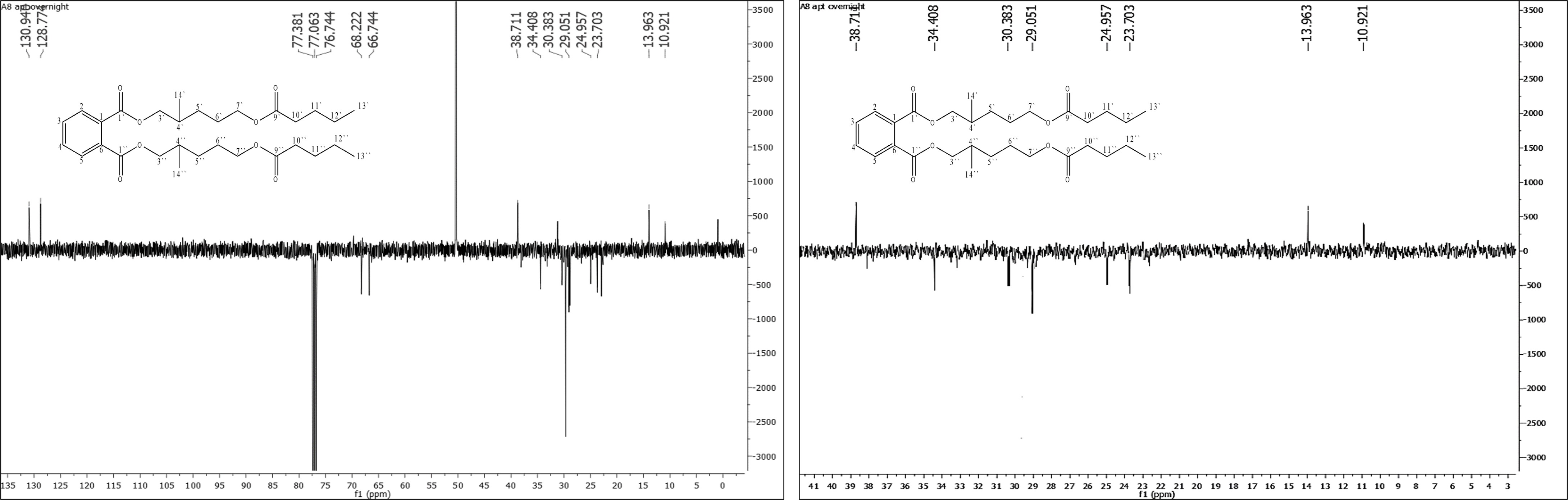

Supplement: S10 Fig — (JPG) [file pone.0313616.s010.jpg]

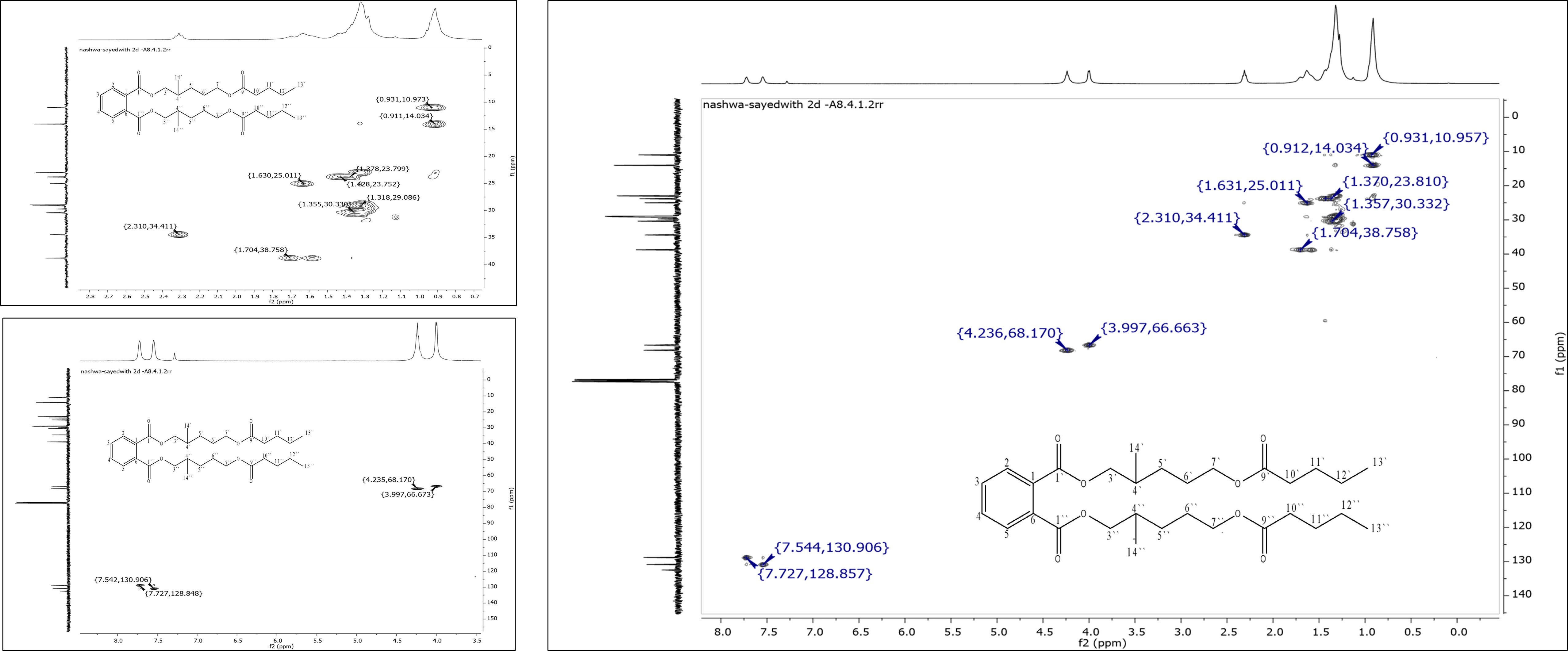

Supplement: S11 Fig — (JPG) [file pone.0313616.s011.jpg]

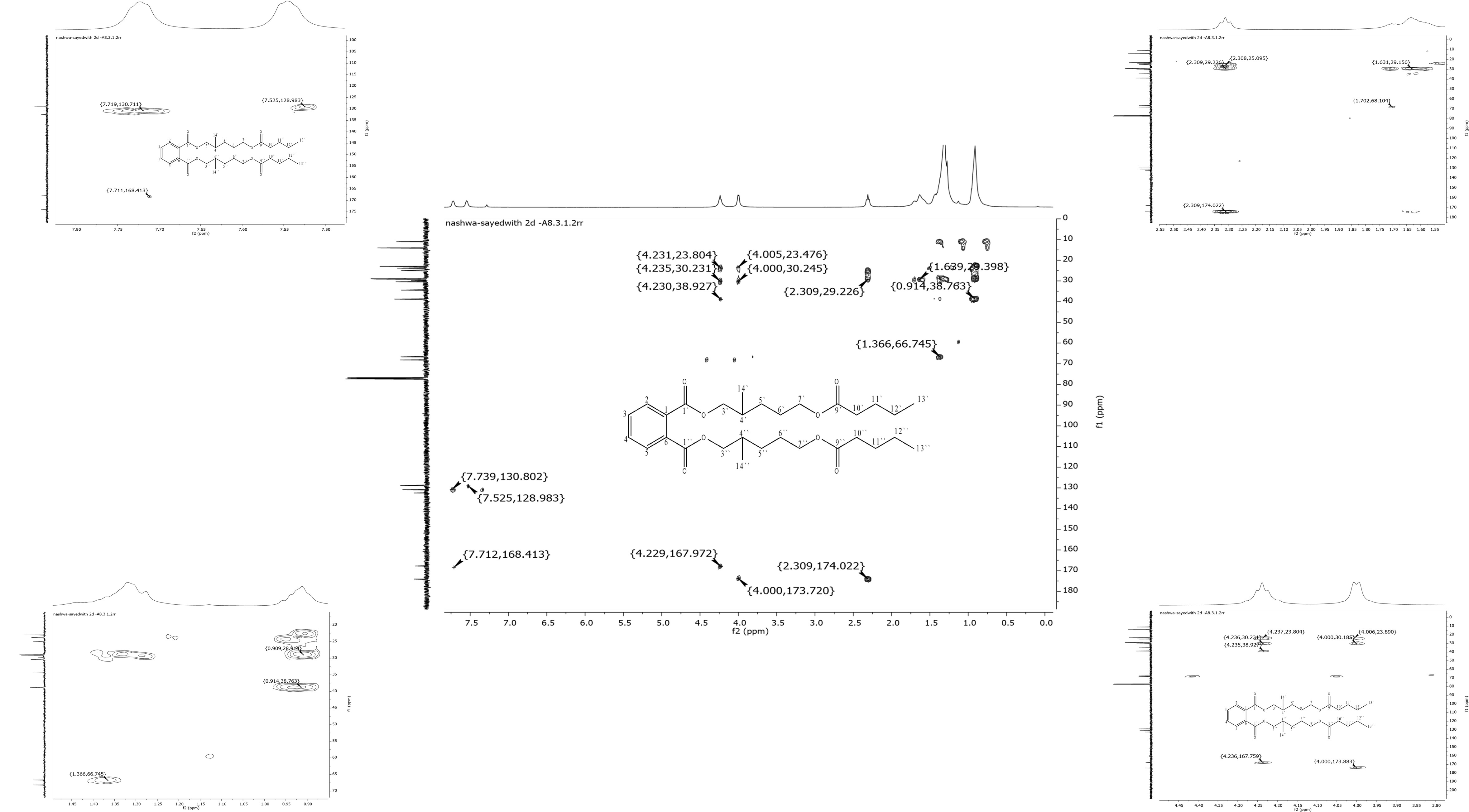

Supplement: S12 Fig — (JPG) [file pone.0313616.s012.jpg]

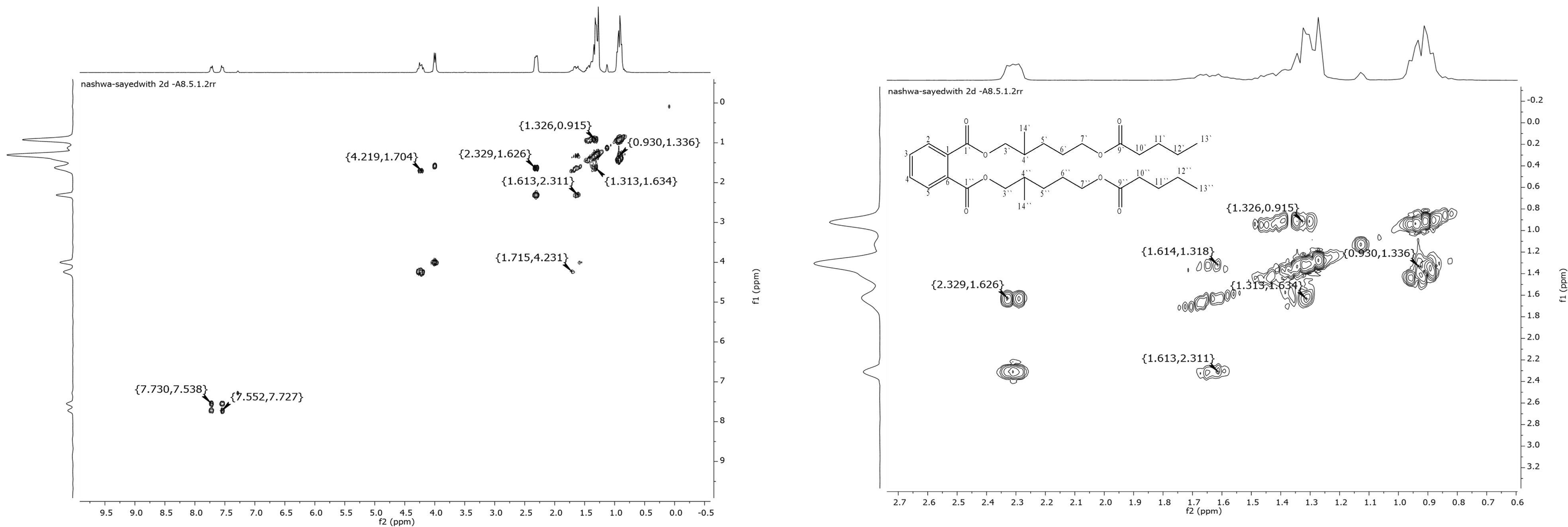

Supplement: S13 Fig — (JPG) [file pone.0313616.s013.jpg]

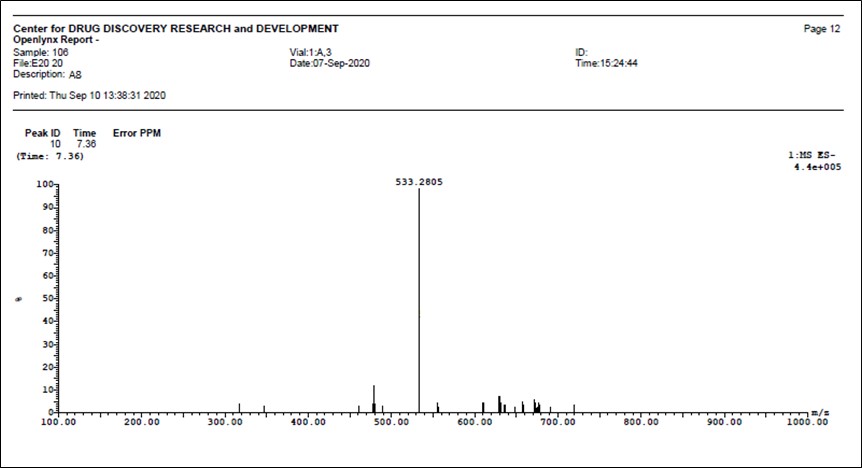

Supplement: S14 Fig — (JPG) [file pone.0313616.s014.jpg]

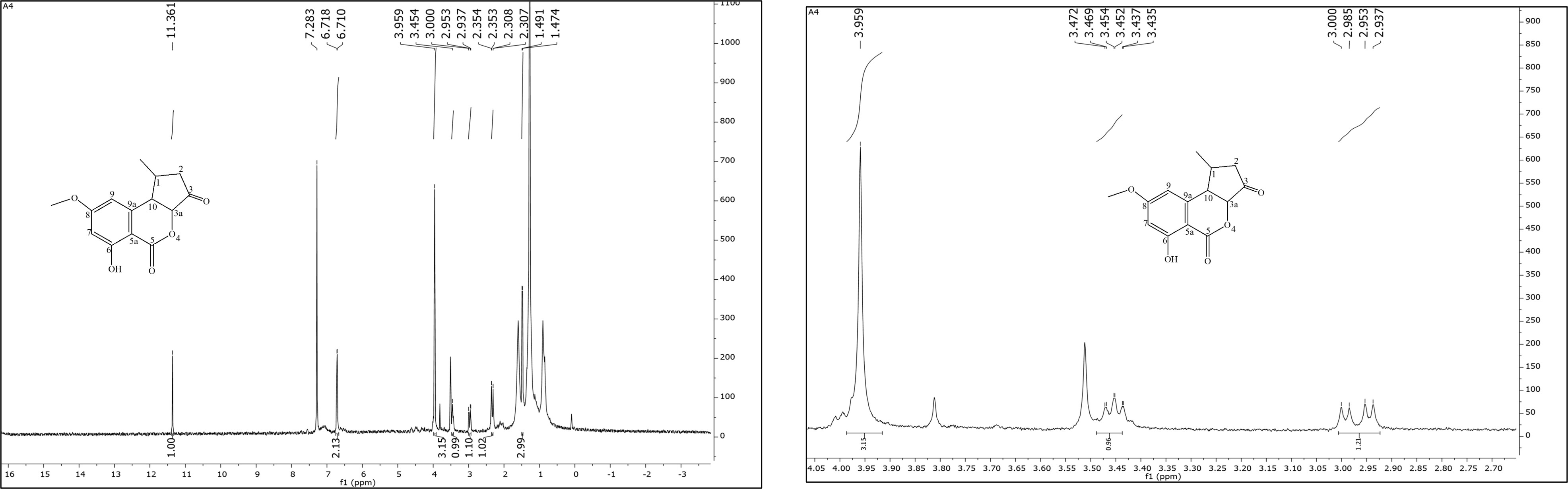

Supplement: S15 Fig — (JPG) [file pone.0313616.s015.jpg]

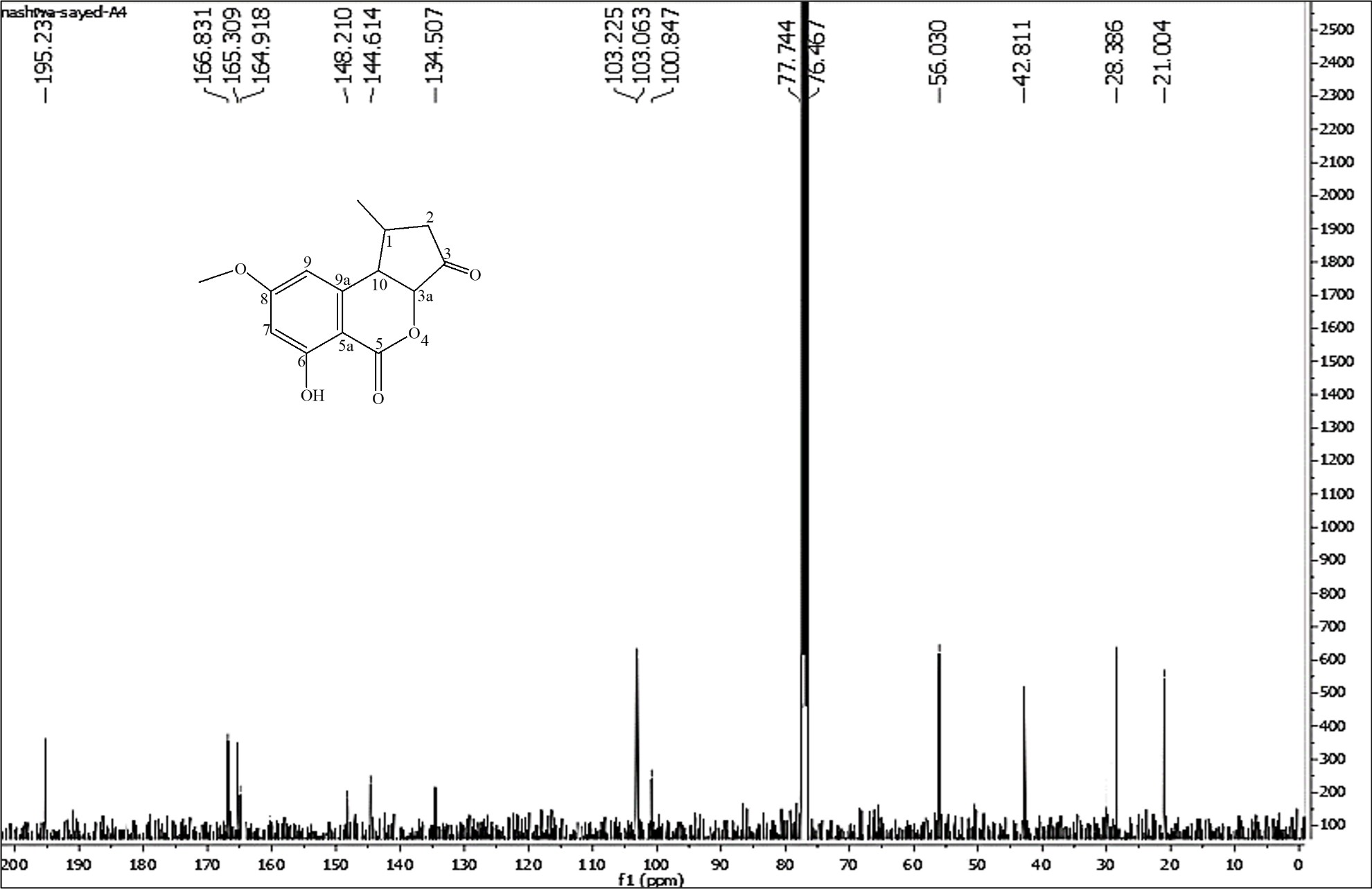

Supplement: S16 Fig — (JPG) [file pone.0313616.s016.jpg]

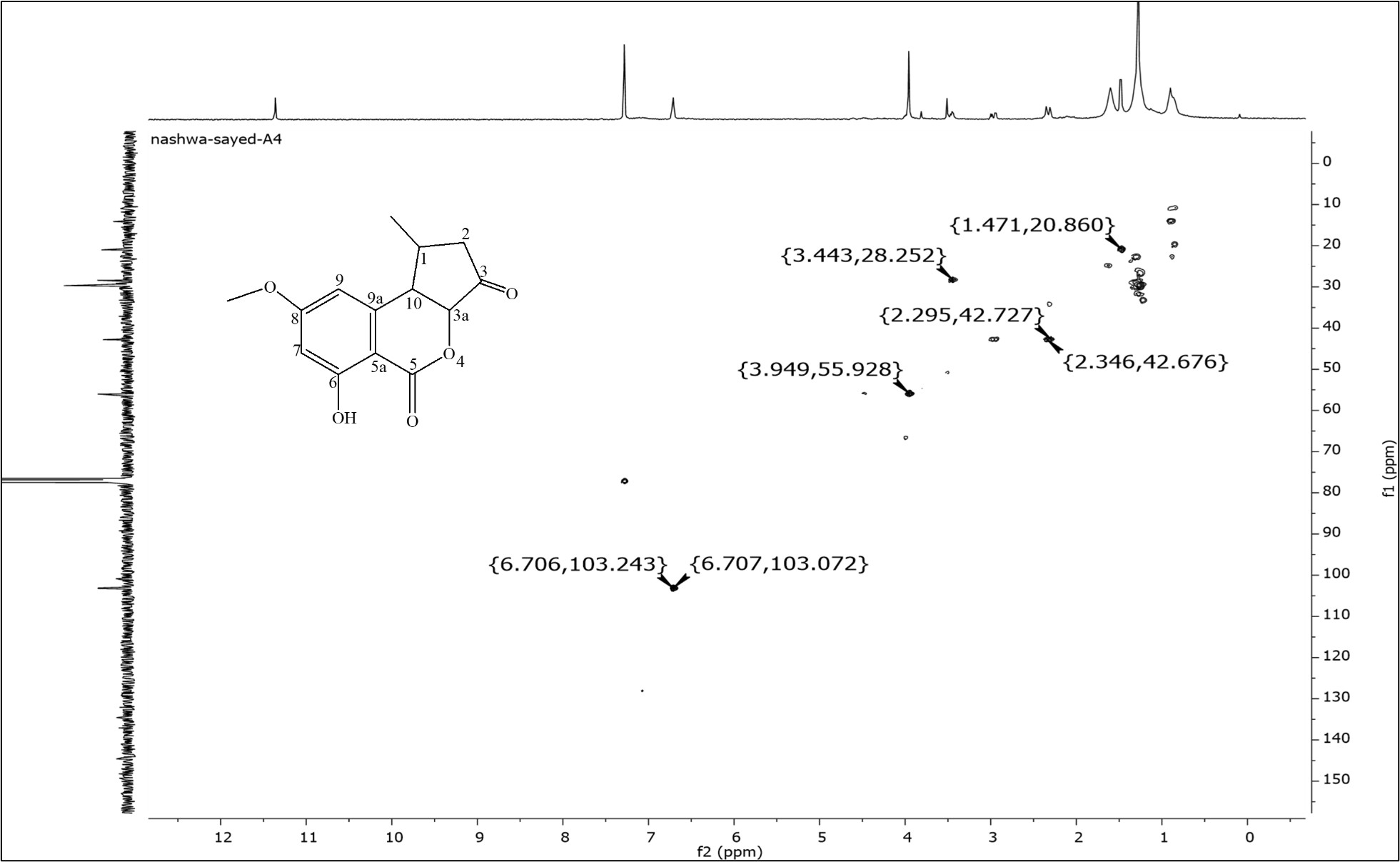

Supplement: S17 Fig — (JPG) [file pone.0313616.s017.jpg]

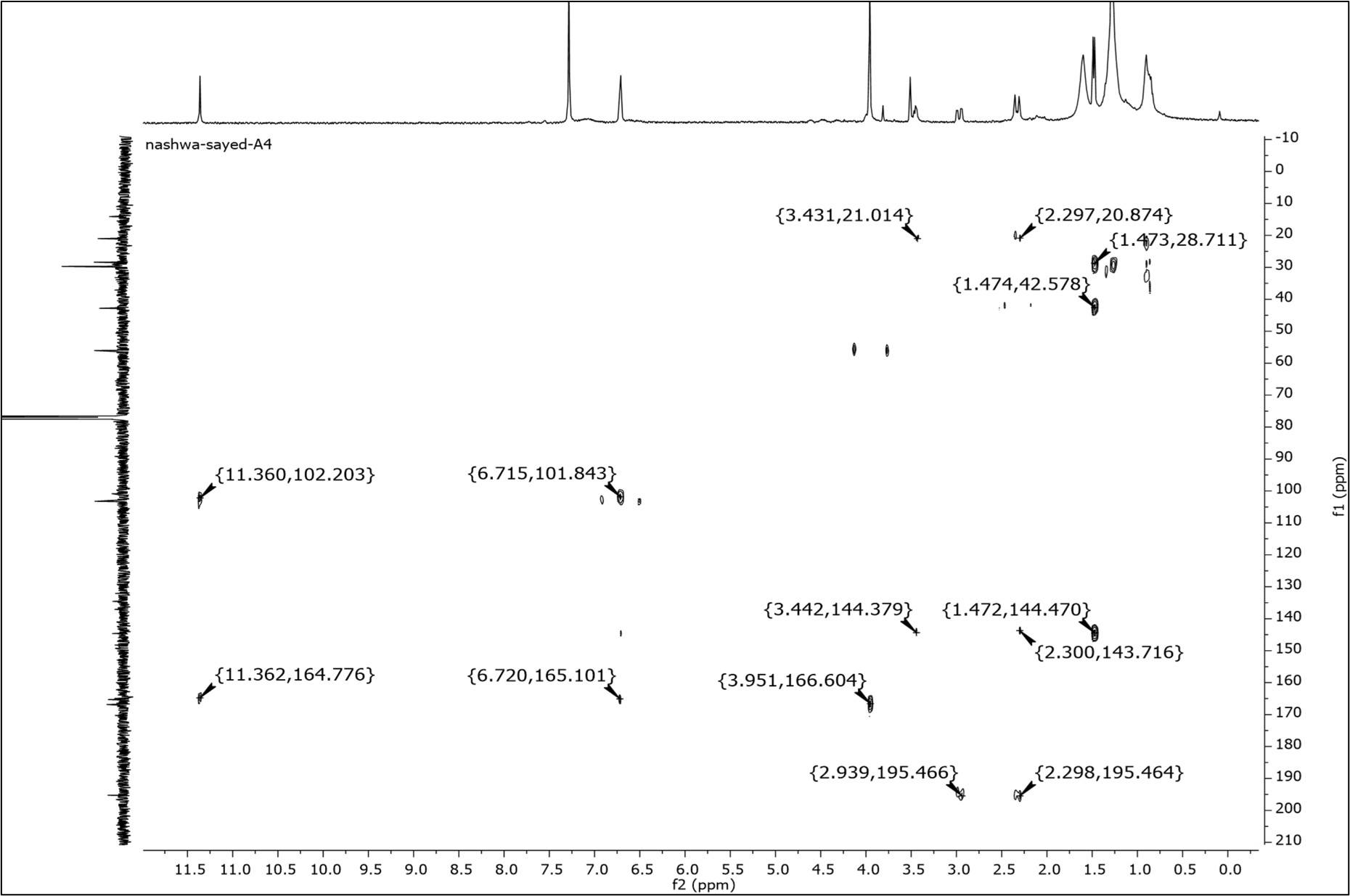

Supplement: S18 Fig — (JPG) [file pone.0313616.s018.jpg]

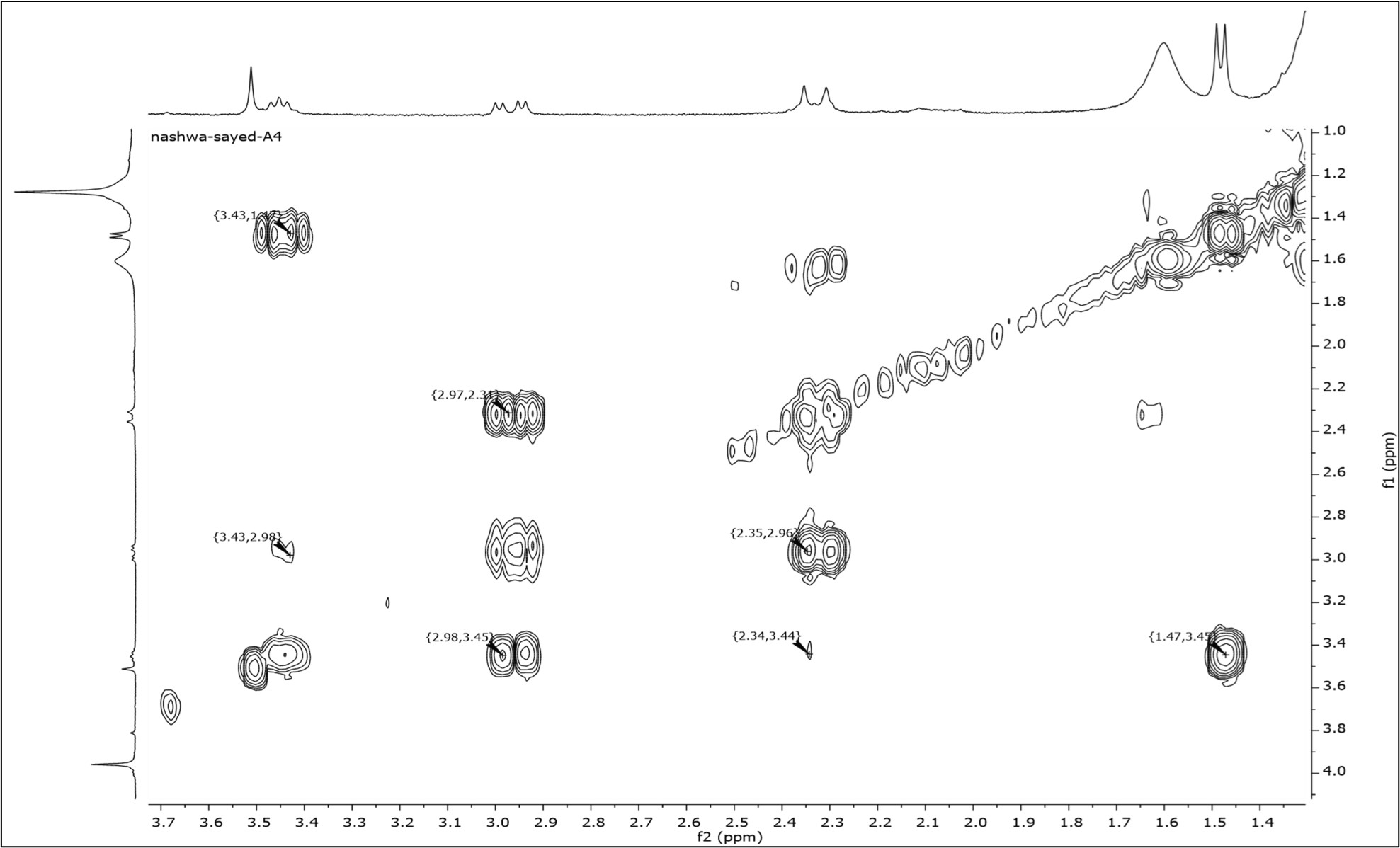

Supplement: S19 Fig — (JPG) [file pone.0313616.s019.jpg]

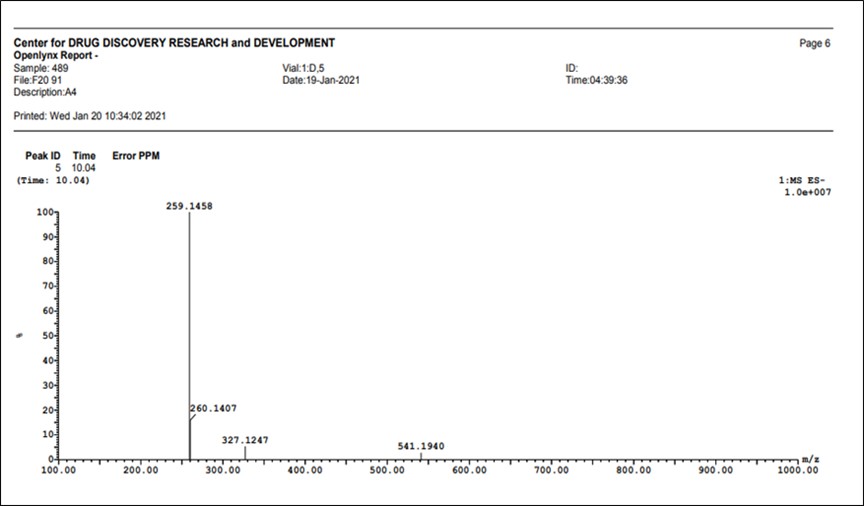

Supplement: S20 Fig — (JPG) [file pone.0313616.s020.jpg]

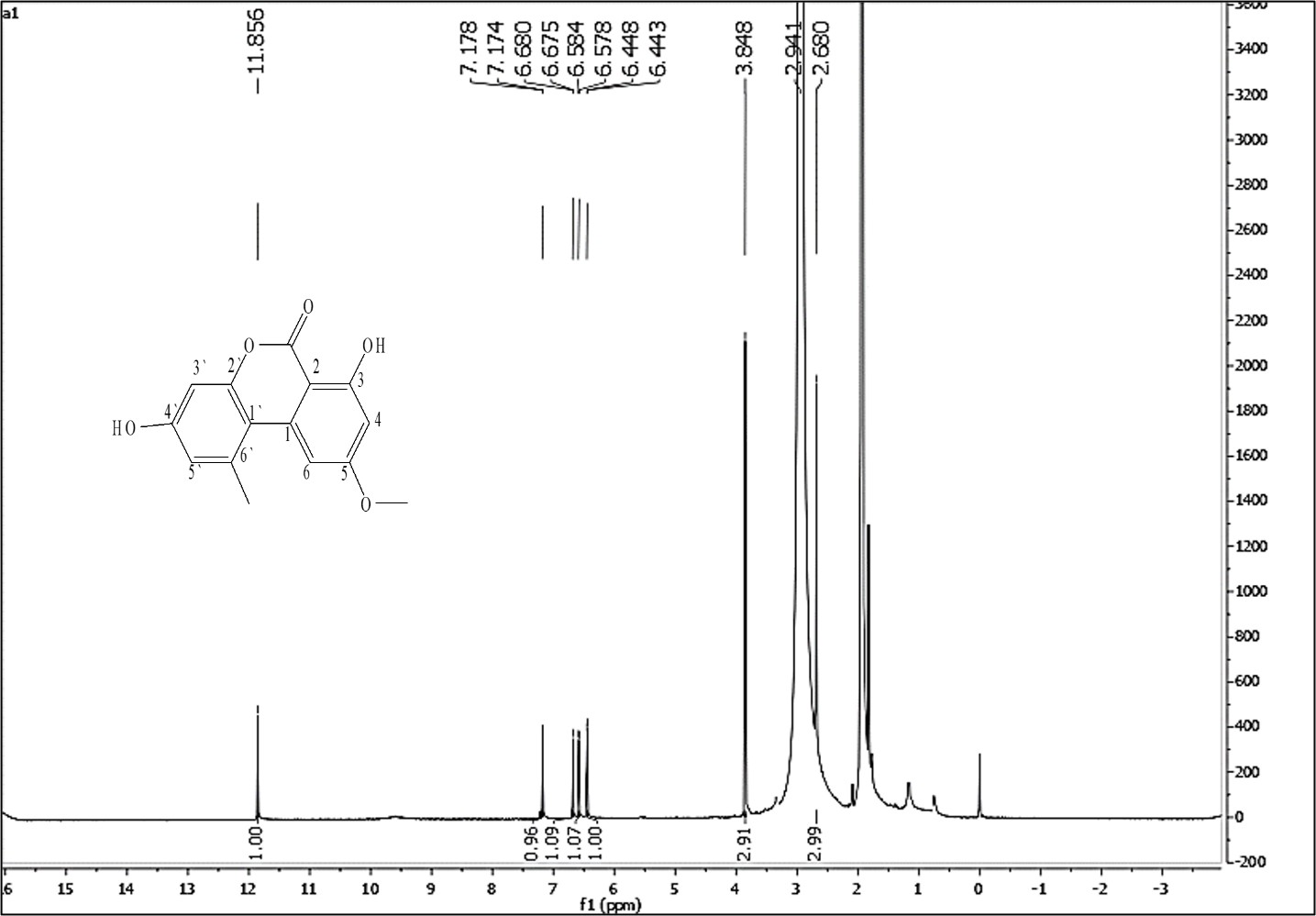

Supplement: S21 Fig — (JPG) [file pone.0313616.s021.jpg]

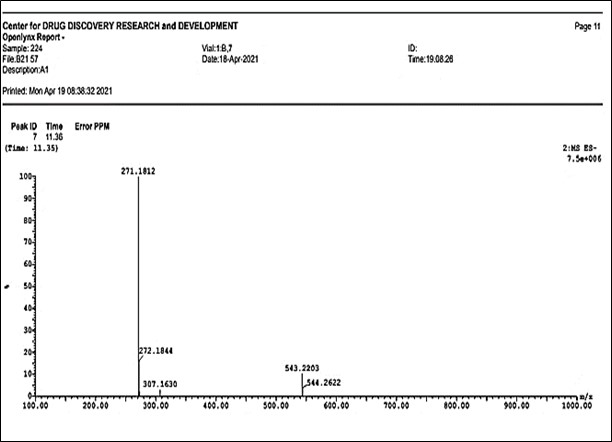

Supplement: S22 Fig — (JPG) [file pone.0313616.s022.jpg]

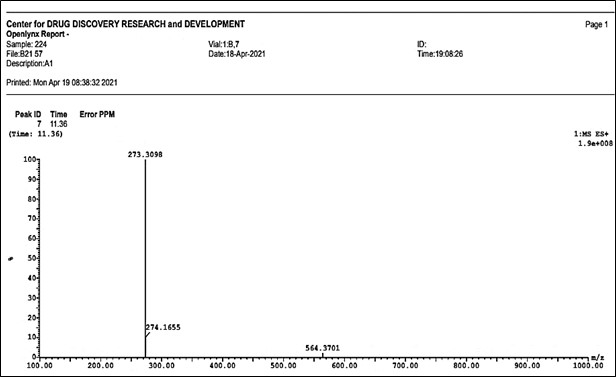

Supplement: S23 Fig — (JPG) [file pone.0313616.s023.jpg]

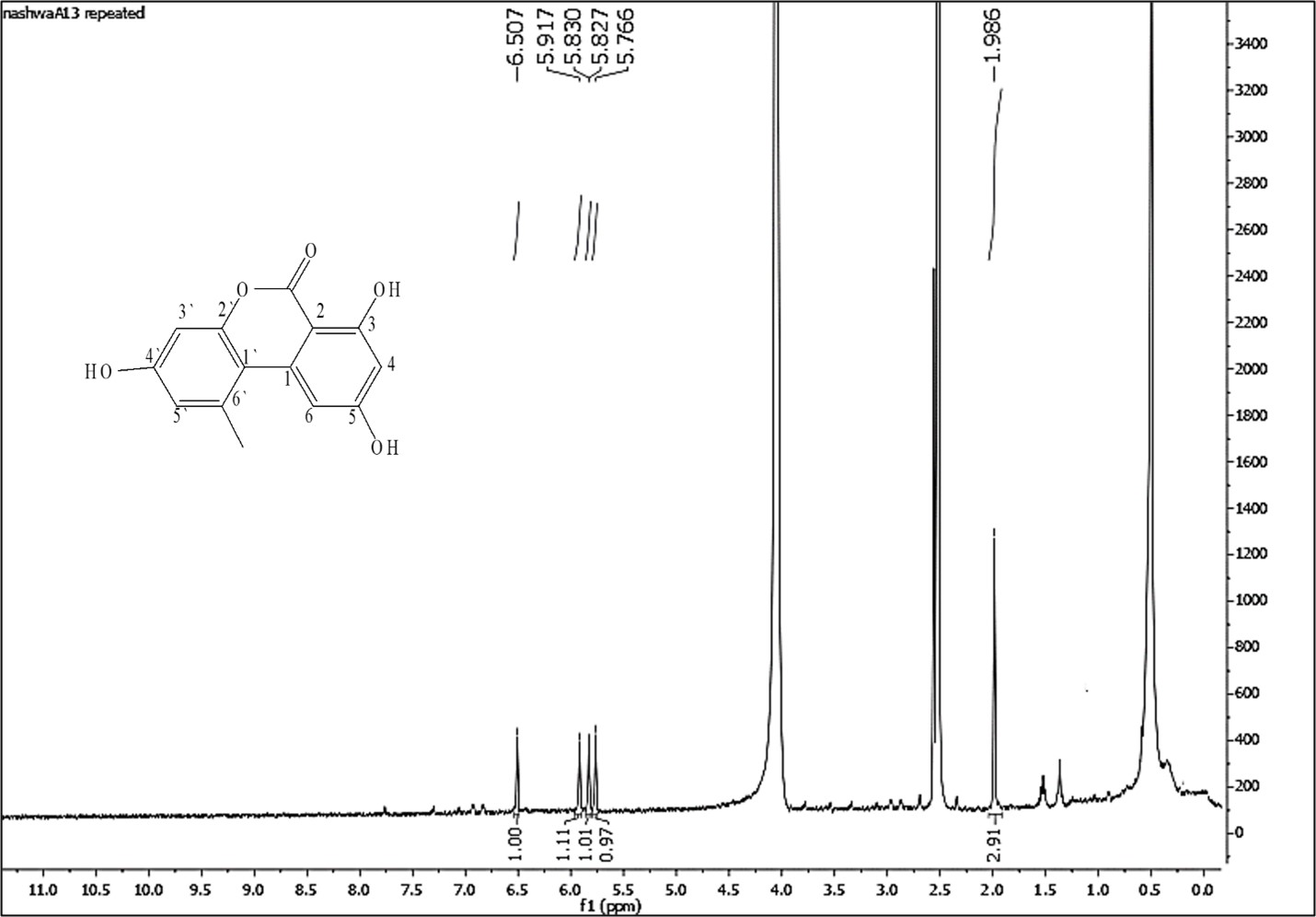

Supplement: S24 Fig — (JPG) [file pone.0313616.s024.jpg]

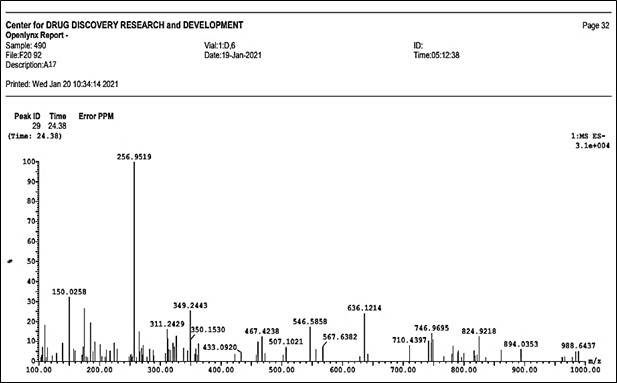

Supplement: S25 Fig — (JPG) [file pone.0313616.s025.jpg]

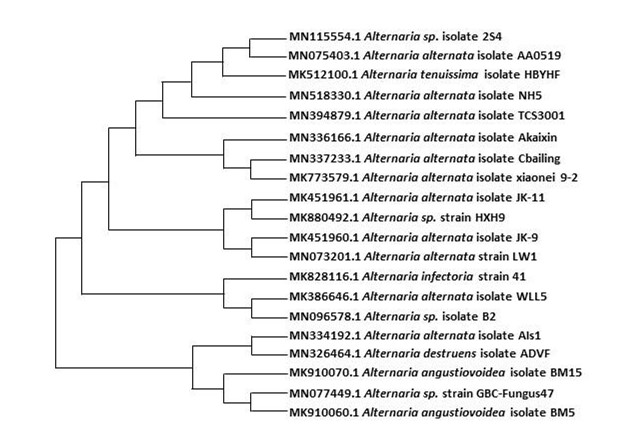

Supplement: S26 Fig — (JPG) [file pone.0313616.s026.jpg]

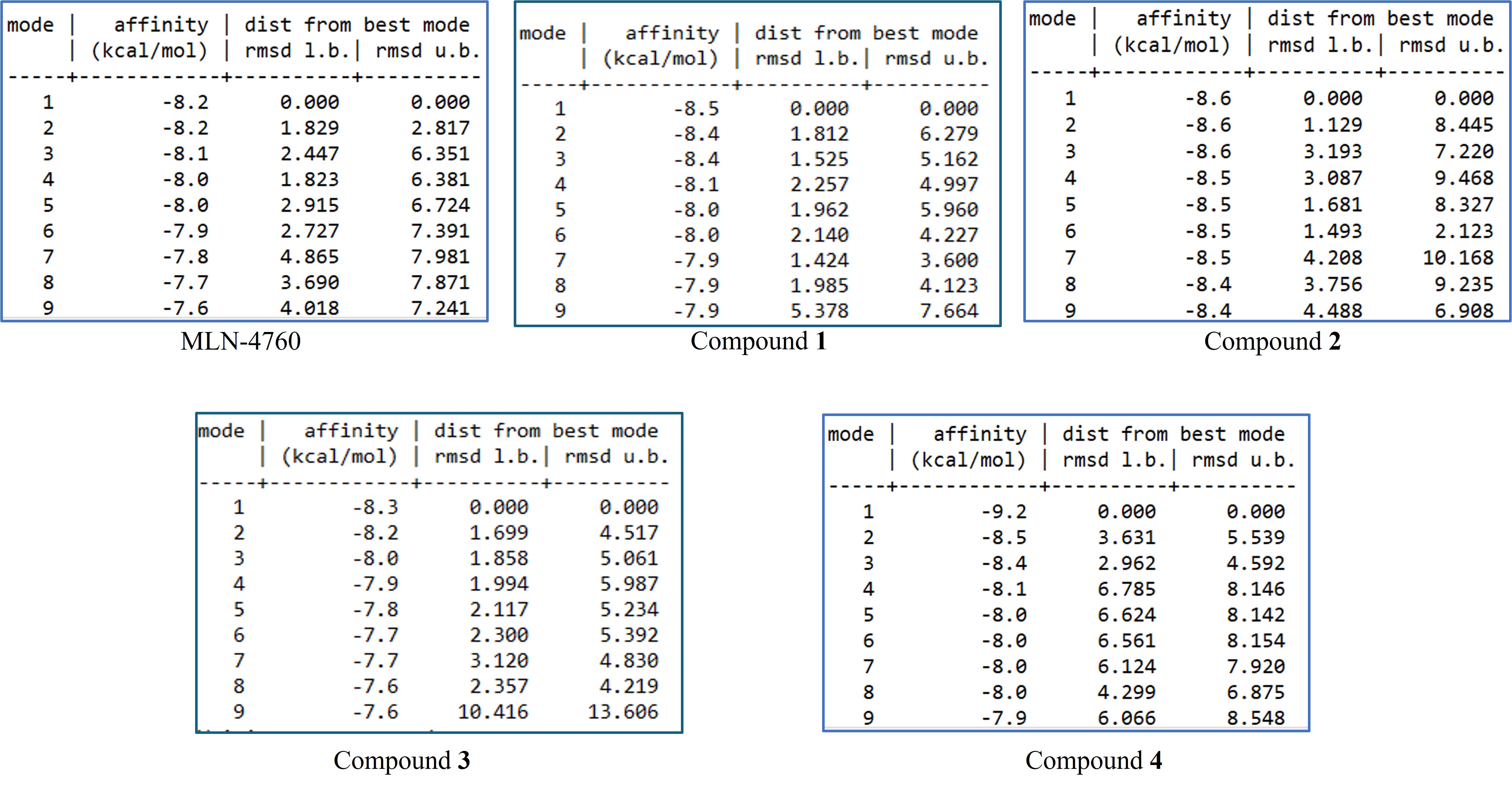

Supplement: S27 Fig — (JPG) [file pone.0313616.s027.jpg]

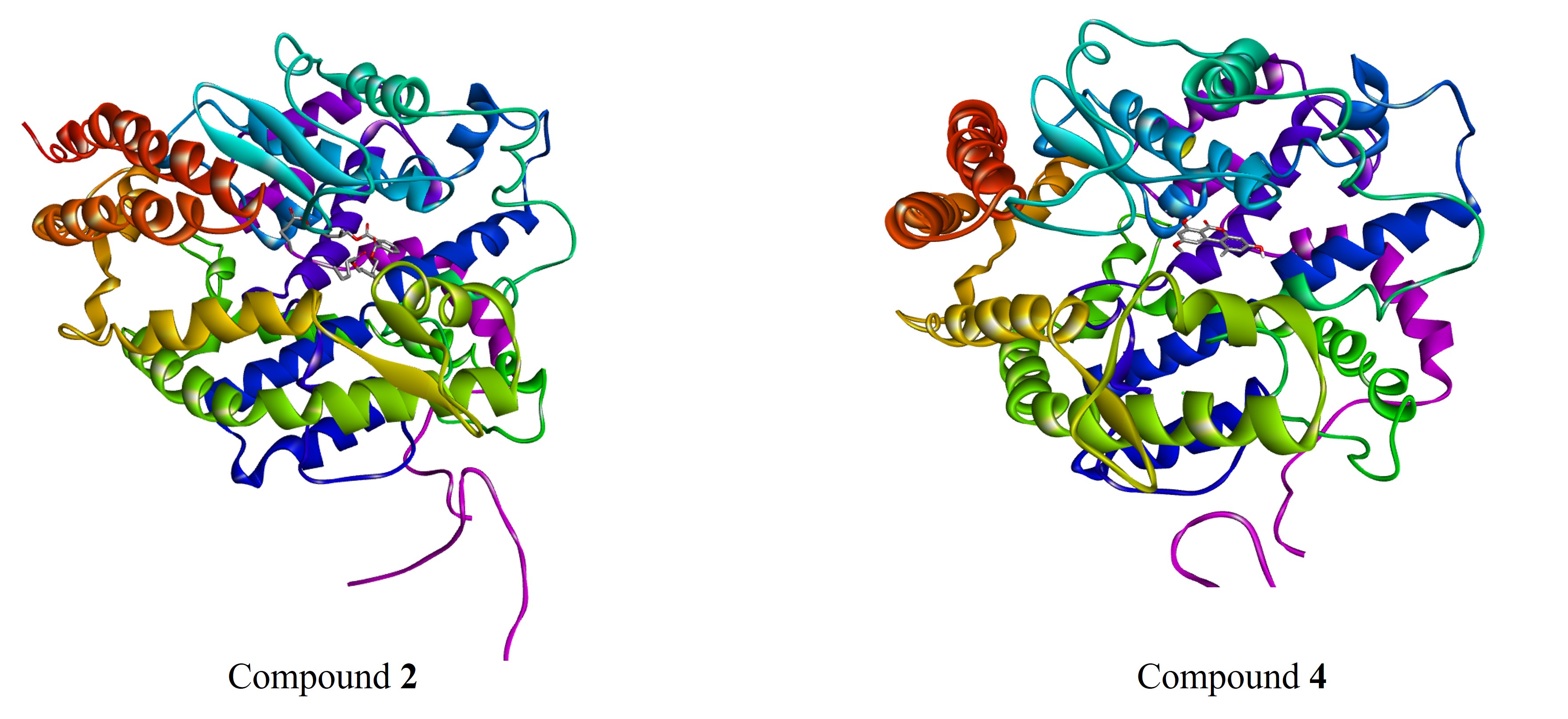

Supplement: S28 Fig — (JPG) [file pone.0313616.s028.jpg]

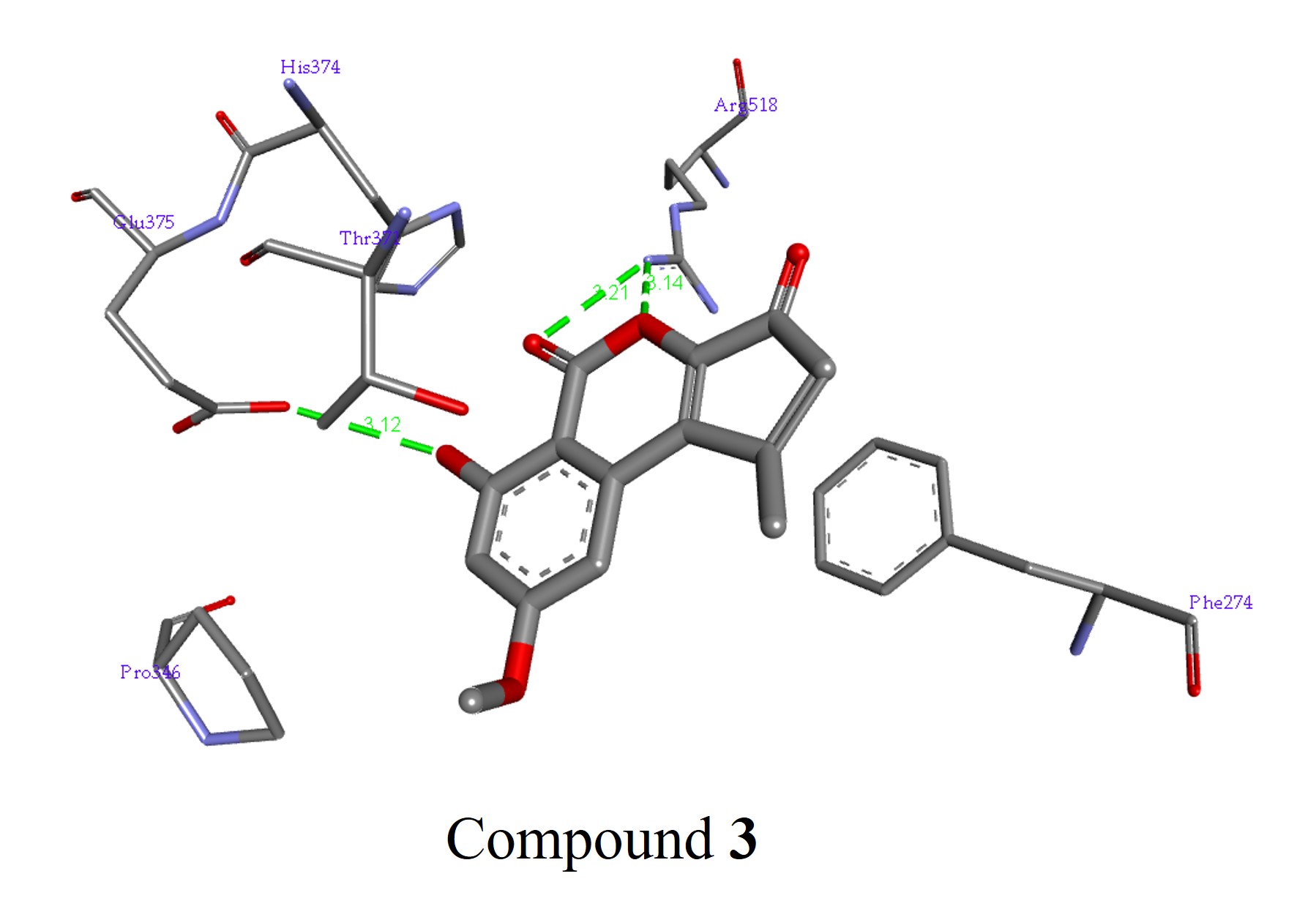

Supplement: S29 Fig — (JPG) [file pone.0313616.s029.jpg]

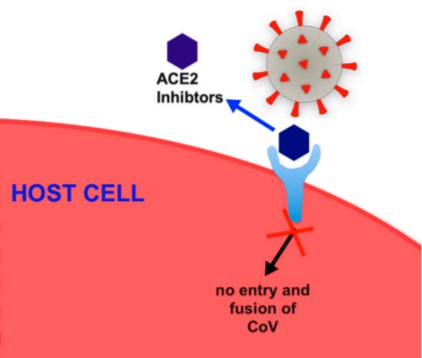

Supplement: S30 Fig — (JPG) [file pone.0313616.s030.jpg]

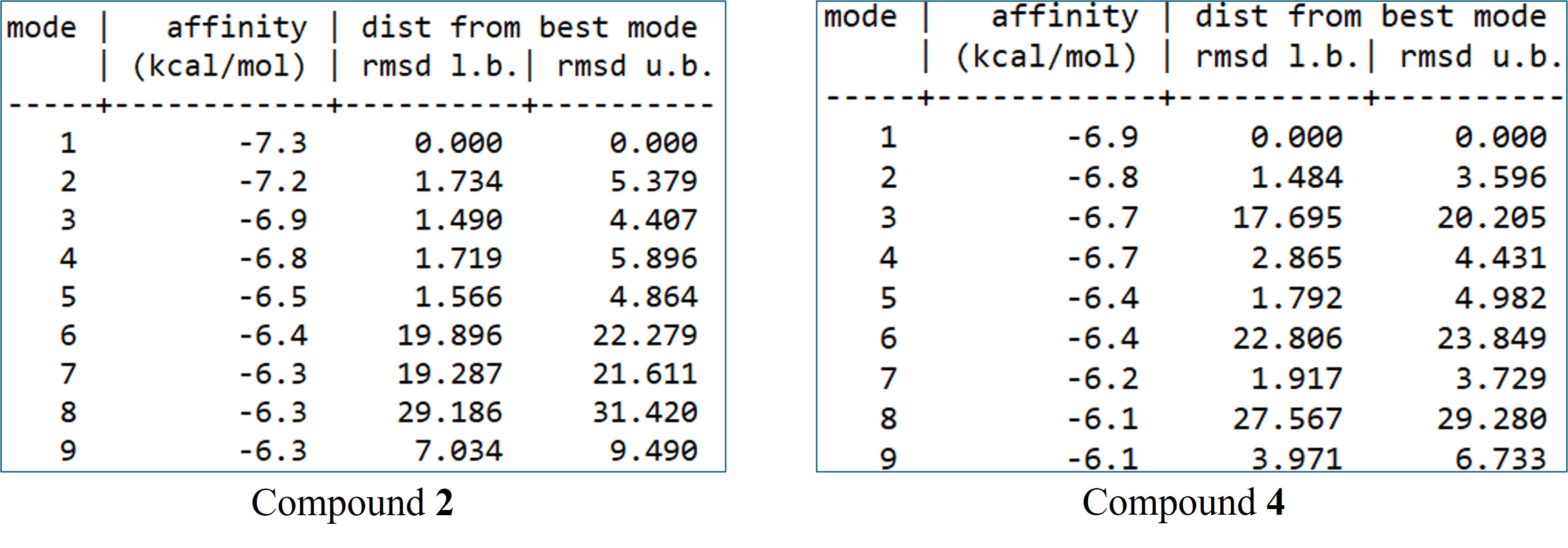

Supplement: S31 Fig — (JPG) [file pone.0313616.s031.jpg]

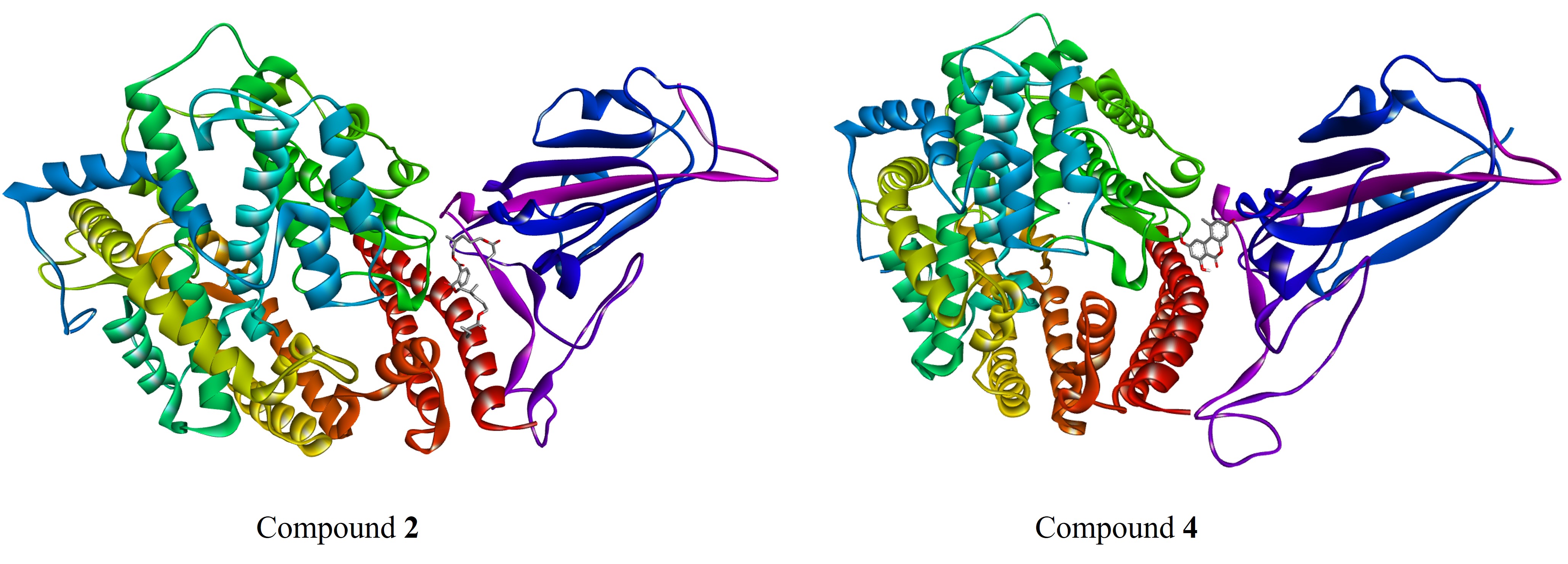

Supplement: S32 Fig — (JPG) [file pone.0313616.s032.jpg]

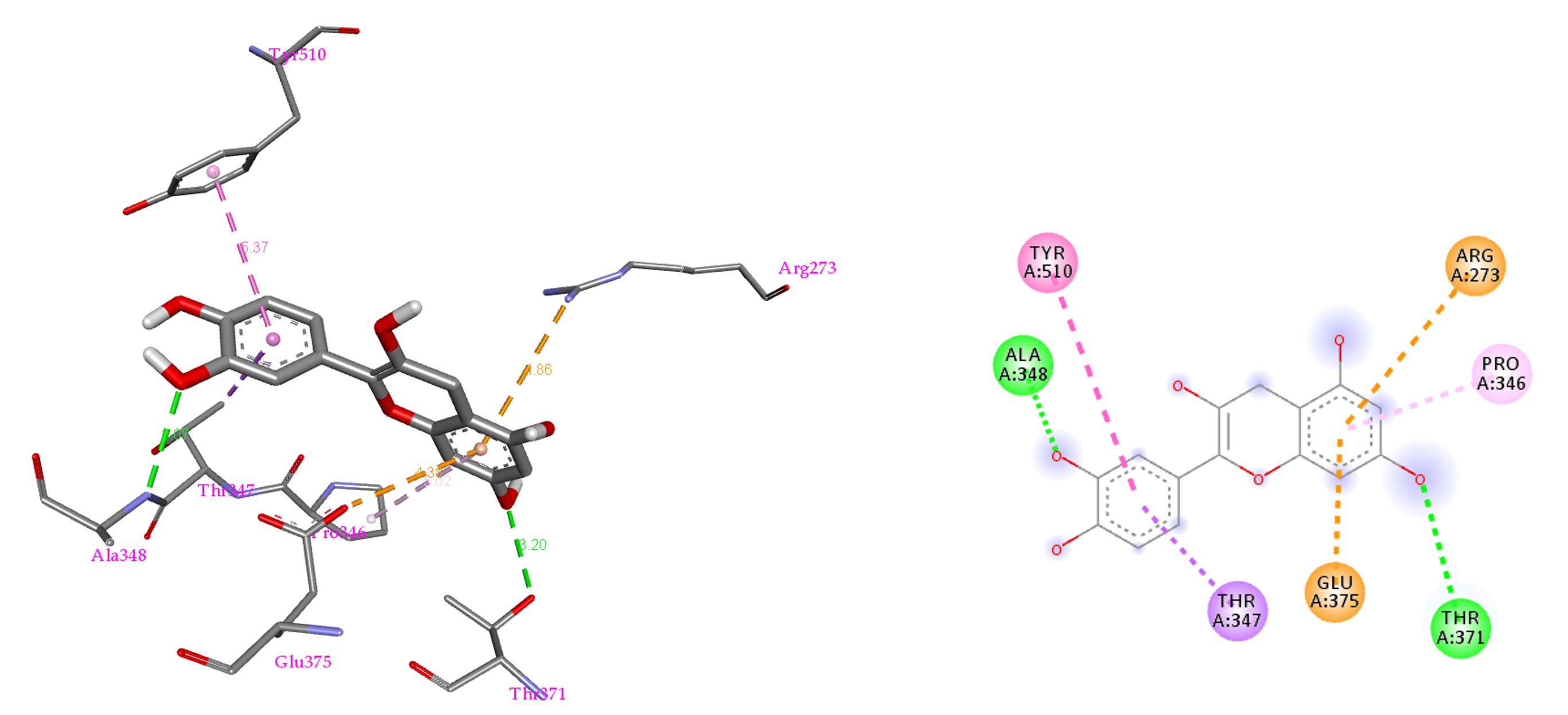

Supplement: S33 Fig — Docking of Quercetin inside the binding site of SARS-Cov-2 S protein RBD in complex with ACE2. (Quercetin was docked into the binding interface between the ACE2 and viral (S) RBD and 9 possible conformers were obtained with good binding affinity (ranging from -8.9 to -7.9 kcal/mol)). (JPG) [file pone.0313616.s033.jpg]

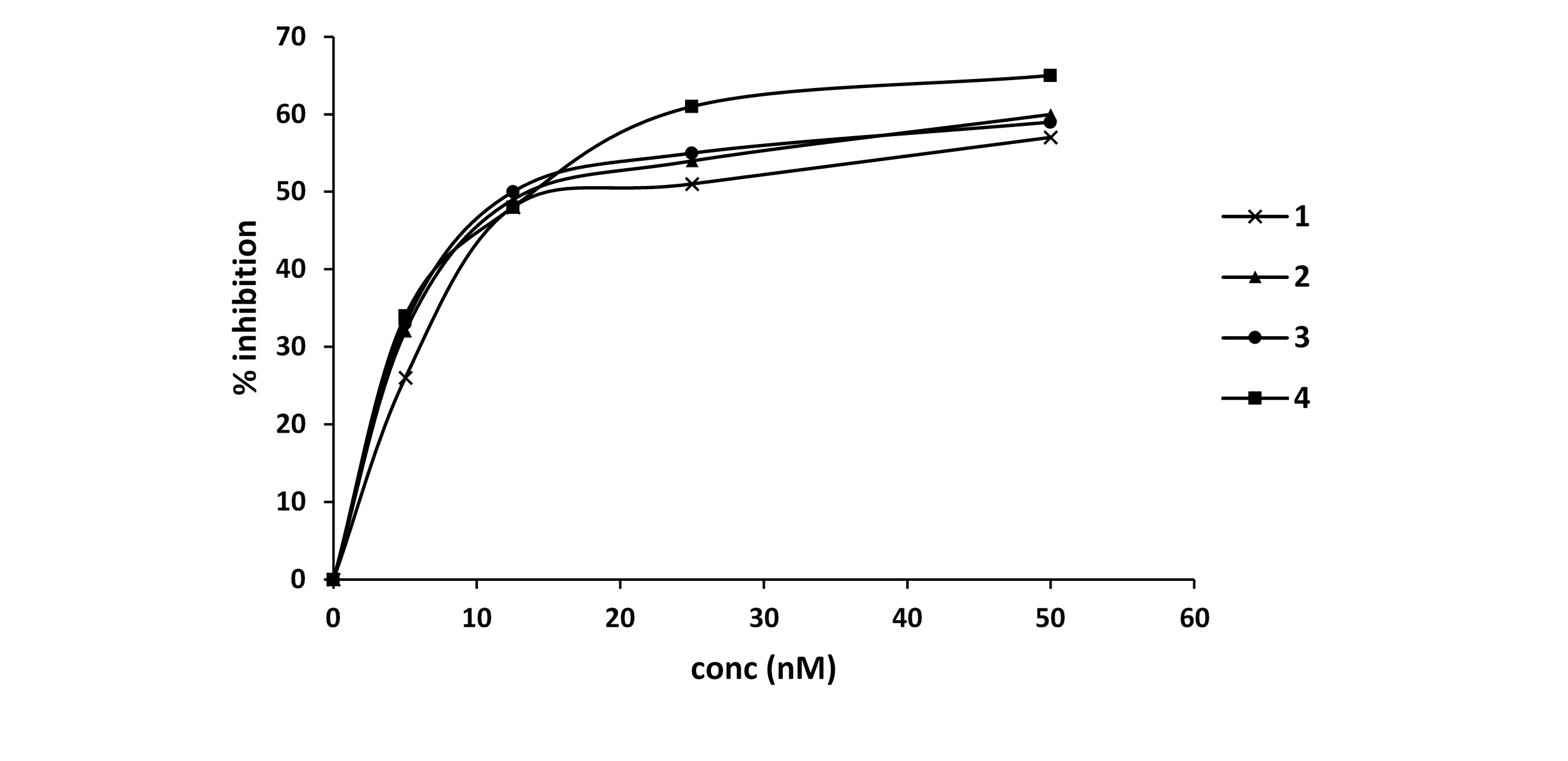

Supplement: S34 Fig — Values represent % ACE2 inhibition (mean ± SD) of three replicates. (JPG) [file pone.0313616.s034.jpg]

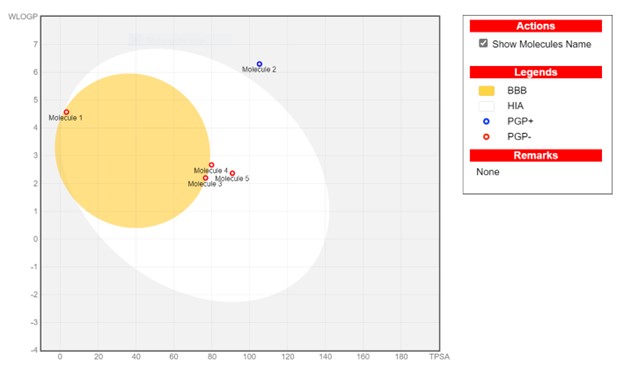

Supplement: S35 Fig — (JPG) [file pone.0313616.s035.jpg]

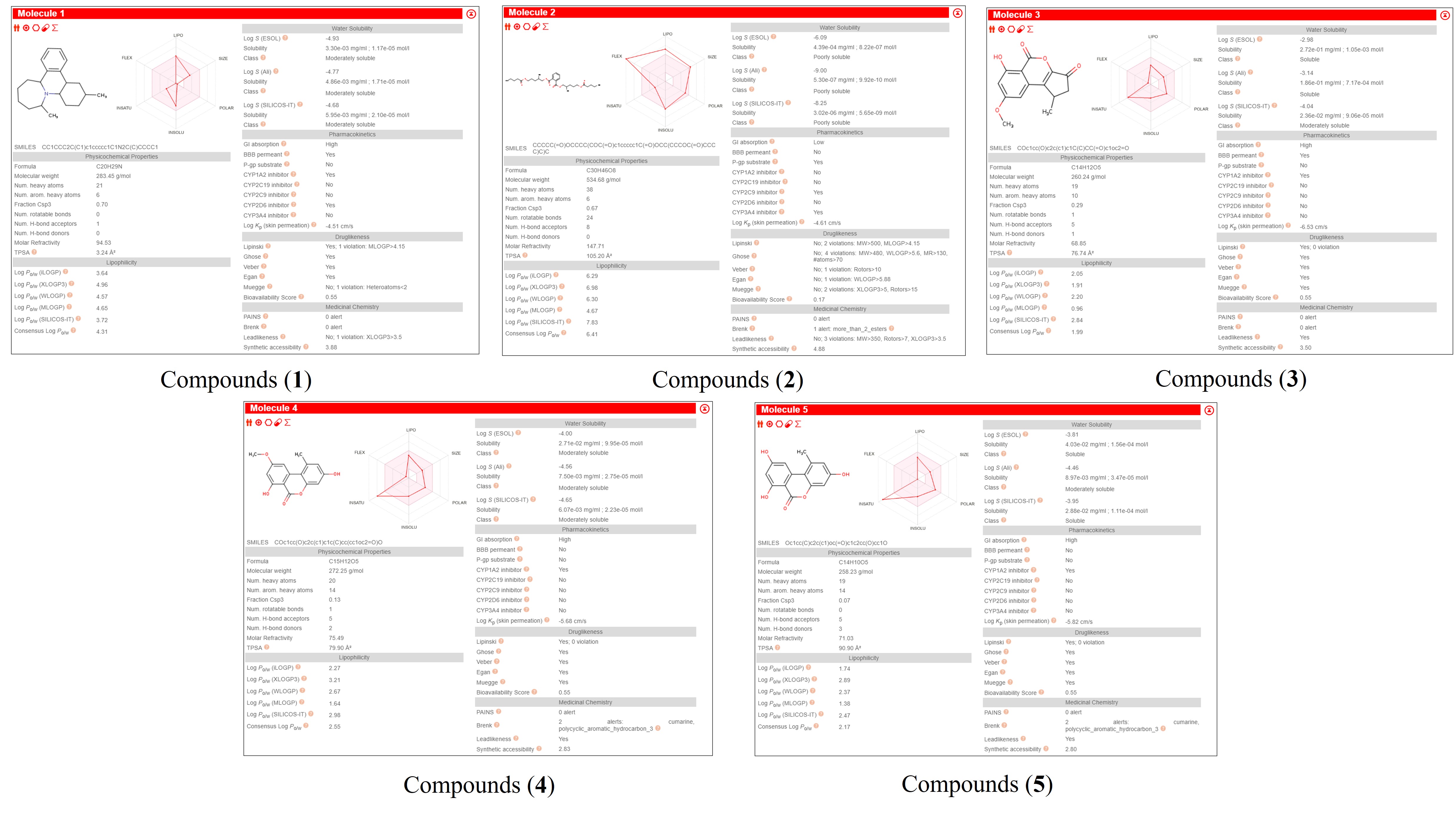

Supplement: S36 Fig — (JPG) [file pone.0313616.s036.jpg]

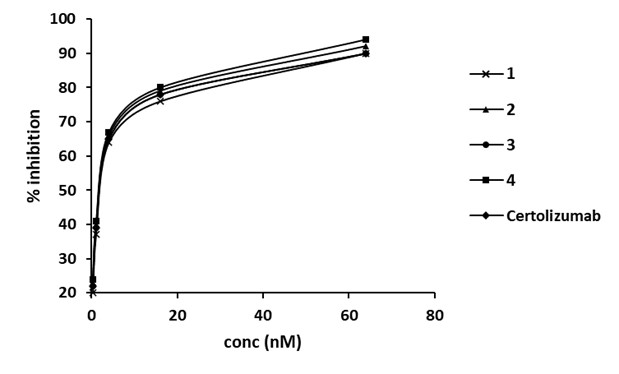

Supplement: S37 Fig — Values represent % TNF-α inhibition (mean ± SD) of three replicates. (JPG) [file pone.0313616.s037.jpg]

ACE2

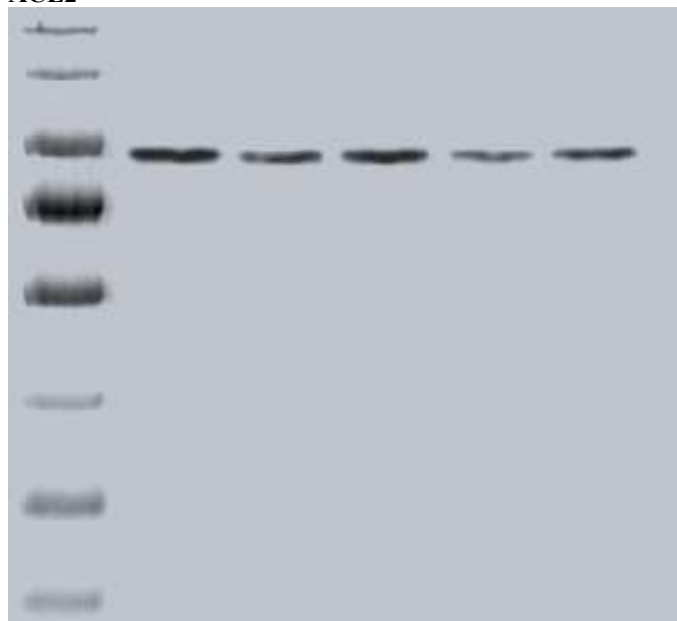

Supplement: S1 Raw images — (PDF) [file pone.0313616.s040.pdf]
