# Supplementary material for: Secondary metabolites of Alternaria alternate appraisal of their SARS-CoV-2 inhibitory and anti-inflammatory potentials
Source: PLoS One. 2025 Jan 24;20(1):e0313616. doi: 10.1371/journal.pone.0313616 (PMC11760621; doi:10.1371/journal.pone.0313616)
Supplement: S1 Table — (DOCX) [file pone.0313616.s038.docx]

**S1 Table:**  ***TNF-α, IL-6* and *β-actin* primer sequences for RT-PCR**.

| **Gene** | **Primer** | **Sequence** |
| --- | --- | --- |
| ***TNF-α*** | Forward  Reverse | 5’-CTCTTCTGCCTGCTGCACTTTG-3’  5'-ATGGGCTACAGGCTTGTCACTC-3' |
| ***IL-6*** | Forward  Reverse | 5’-AGACAGCCACTCACCTCTTCAG-3’  5'-TTCTGCCAGTGCCTCTTTGCTG-3' |
| ***β-actin*** | Forward  Reverse | 5’-GCACCACACCTTCTACAATG-3’  5’-TGCTTGCTGATCCACATCTG-3’ |
