# Supplementary material for: Secondary metabolites of Alternaria alternate appraisal of their SARS-CoV-2 inhibitory and anti-inflammatory potentials
Source: PLoS One. 2025 Jan 24;20(1):e0313616. doi: 10.1371/journal.pone.0313616 (PMC11760621; doi:10.1371/journal.pone.0313616)
Supplement: S2 Table — (DOCX) [file pone.0313616.s039.docx]

**S2 Table: In silico study, describe briefly the binding affinity, binding amino acids, bond length.**

| **Compound** | **Protein** | **Binding Affinity Range**  **(kcal/mol)** | **Non-covalent Interactions**  **(Å)** |
| --- | --- | --- | --- |
| **2** | *h*ACE2 | -8.6 to -8.4 | 1) Two H-bonds with Thr371 (2.99 and 3.04 Å, respectively) His345 (3.06 Å) and Ala348 (3.16Å); 2) hydrophobic interaction with Tyr127 (5.05 Å), Phe274 (4.72 Å), Cys344 (5.20 Å), Thr347 (4.55 Å), His374 (4.82 Å), and Tyr515 (5.01 Å) |
| **4** | *h*ACE2 | -9.2 to -7.9 | 1) H-bonds with Glu375 (3.21Å), Thr445 (2.70Å), and Arg518 (3.08Å); 2) hydrophobic interaction with Arg273 (4.55 Å), Phe274 (4.83 Å), Pro346 (4.71 Å), and Thr376 (4.55 Å) |
| **2** | SARS-CoV-2 spike receptor binding domain complexed with ACE2 | -7.3 to -6.3 | 1) H-bonds with Asn33 (3.15 Å), Phe390 (2.94 Å), Arg393 (2.88 Å and 3.08 Å), Arg403 (2.81 Å) and Tyr505 (2.97 Å); 2) Hydrophobic interactions with His34 (4.40 Å) and Val193 (4.73 Å). |
| **4** | SARS-CoV-2 spike receptor binding domain complexed with ACE2 | –6.9 to -6.1 | 1) H-bonds with His34 (3.19 Å), Arg393 (2.95 Å), Glu406 (2.87 Å), Arg408 (3.04 Å), Gln (2.94 Å) and Tyr505 (2.96 Å); 2) Hydrophobic interactions with Ala386 (4.52 Å) and Arg403 (4.72 Å). |
